# Effects of solvent-based adhesive removal on the subsequent dual analysis of fingerprint and DNA

**DOI:** 10.1007/s00414-023-03042-w

**Published:** 2023-07-04

**Authors:** Christian Gausterer, Gerald Birnbaumer, Wolfgang Ondrovics, Christina Stein

**Affiliations:** 1 FDZ-Forensisches DNA Zentrallabor GmbH, Medical University of Vienna, Sensengasse 2, 1090 Vienna, Austria; 2 Criminal Intelligence Service Austria, Department II/BK/6 Forensics, Unit II/BK/6.3.1 – Dactyloscopic Reference Laboratory, Josef-Holaubek-Platz 1, 1090 Vienna, Austria; 3 Criminal Intelligence Service Austria, Department II/BK/6 Forensics, Sub Department II/BK/6.3 – Crime Scene, Roßauer Lände 5, 1090 Vienna, Austria

**Keywords:** Solvent-based adhesive removal, Adhesive surface, Latent fingerprint development, PCR inhibition, DNA recovery, DNA transfer

## Abstract

The combined approach of classical fingerprinting and DNA profiling is a powerful tool in forensic investigations of latent “touch” traces. However, little attention has been paid to the organic solvents frequently used in dactyloscopic laboratories to facilitate the separation of adhesive evidence prior to fingerprint development and downstream effects on subsequent DNA profiling. In the present study, we tested a selection of adhesive removers (*n* = 9) and assessed their potential impact on DNA recovery and amplification by PCR. Thereby, we identified and characterized novel PCR inhibitors. All investigated chemicals contain volatile organic compounds that evaporate under normal indoor atmospheric conditions. Exposure to certain solvents resulted in increased DNA degradation, but only if evaporation was prevented. A series of adhesive-removal experiments were conducted with prepared mock evidence (self-adhesive postage stamps affixed to paper envelope) to investigate the impact of treatment time and the location of applied traces on DNA recovery and dactyloscopy, respectively. Due to the early onset of print decomposition, we found that only a short treatment time was compatible with the development of fingerprints on the adhesive side of a stamp. Solvents also removed DNA from the adhesive surface, thus resulting in a marked shift in the substrate distribution of recovered DNA from the stamp to the envelope, but not in the reverse direction. Furthermore, we observed that treatment with conventional fingerprint reagents lead to a significant reduction in the amounts of DNA recovered from stamps, while the additional use of adhesive removers did not significantly enhance this effect.

**Supplementary Information:**

The online version contains supplementary material available at 10.1007/s00414-023-03042-w.

## Introduction

Fingerprint and DNA are among the most important investigative tools in law enforcement today, because of their robustness, uniqueness and the successful implementation of systems for automated human identification based on these biometric markers [1]. Both types of evidence can help uncover crime connections and reveal serial offenders by comparing leads from different crime scenes, but the information provided is not merely redundant.

Dactyloscopy is considerably one of the oldest forensic disciplines and one of the fastest, most reliable and cost-effective means of biometric human identification [2]. The arrangement of “touch traces” (e.g. grip marks) may provide further clues to the reconstruction of criminal activities. Its molecular composition may provide additional information about chemical contaminants, fingerprint age and the donor (e.g. age and health status) [3]. Depending on the circumstances of a case, the evaluation of dactyloscopic evidence may be less complicated compared to DNA, as transfer is less likely with fingerprints (unless adhesives are involved).

Unlike dactyloscopy, forensic DNA analysis is not limited to a specific type of biological source, and minute amounts of bodily material (e.g. tissue, bone, body fluids, hair, skin particles and sweat) or even “touch” may be sufficient for definitive assignment to its donor [4, 5]. Furthermore, genetic trace evidence may provide additional information for establishing investigative leads that can go well beyond human identification [6] e.g. (distant) kinship relations [7], biogeographic ancestry [8, 9], phenotypic appearance [10], chronological age [11] and typing of non-human species [12]. Thus, depending on the circumstances of a case and the available evidence, it may be appealing to investigate both. Accordingly, several studies have assessed possibilities for the dual analysis of fingerprint and DNA [13–16].

Fingerprints are fragile and can easily be compromised by common DNA collection techniques (e.g. swabbing), which is why a dactyloscopic examination is usually performed prior to DNA analysis [17]. On the other hand, fingerprint development can result in significant losses in DNA quantity and quality, depending on the original sample type, the substrate of deposition and details of sample processing [13, 18]. Previous studies have focused primarily on the core steps of fingerprint development and their downstream effects on DNA typing, ignoring the fact that additional ancillary procedures (e.g. adhesive removal processes) are often used in forensic dactyloscopy.

Adhesives are versatile and ubiquitous in daily life. It is therefore not surprising that adhesive evidence (tapes, postage stamps, labels and films) is often found at crime scenes and subject to forensic investigations. Adhesive surfaces can provide very efficient tools for collecting latent contact traces from porous and non-porous surfaces [19–22].

A common complication in forensic dactyloscopy regarding adhesive evidence is hindered access to a trace of interest (e.g. a fingerprint located on the sticky side of a postage stamp affixed to an envelope). Ridge details of a fingerprint can be easily impaired making them unsuitable for human identification. To make matters worse, the behaviour of the adhesive layers during detachment can be difficult to predict due to differences in material properties, product quality, unknown storage conditions and preservation state. Consequently, there is an immanent risk of inefficient and incomplete separation, perhaps leading to fragmentation of adhesive layers and destruction of the enclosed print. Thus, the main challenge is not merely to physically separate adhering objects, but rather to make the hidden trace accessible for analysis without losing relevant information. To maximize fingerprint recovery, a sequential approach to adhesive removal has been recommended [23, 24]. First, rather non-destructive procedures (pulling with tweezers at room temperature) should be used. If these initial attempts fail, further techniques with potentially greater impact (e.g. cooling, solvent and heating) may be applied. The aim is to avoid unnecessary risks to the integrity of the examined traces. In addition, it would be advisable to practice these methods first with similar materials (e.g. stamp and envelope). Solvent-based adhesive removal is effective at separating commonly found pressure sensitive adhesives from all surfaces. It involves the local application of a chemical agent that softens and dissolves the adhesive layers so that the adherent object can be physically lifted from the substrate [24].

In the forensic literature, adhesive removers are mainly discussed in the context of fingerprint development [25, 26], while potential effects on subsequent DNA analysis have received little attention. Spear et al. [27] reported that the combination of adhesive remover (Un-Du) and fingerprint powder on the adhesive side of tape interferes with subsequent PCR-based DNA typing of treated bloody fingerprints. Recently, Ruprecht et al. [15] published a novel approach for the parallel evaluation of dactyloscopic and DNA trace material originating from the same evidence (fingerprint transferred from the sticky side of a postage stamp to the envelope) which involves a step of solvent-based adhesive neutralization and detachment, but potential downstream effects of this treatment step were not investigated in detail.

The aim of our present study was to investigate the impact of solvent-based adhesive removal procedures on some critical steps of forensic DNA analysis, focusing on DNA recovery, transfer and amplification by PCR.

PCR plays a central role in forensic DNA analysis, as it allows the specific detection, amplification and typing of nucleotide polymorphisms starting from a few molecules of genetic material. Efficient amplification requires high DNA polymerase activity and favourable conditions for nucleic acid interactions (template denaturation and primer annealing). Thus, any compound that interferes with the availability and functioning of critical factors can be an inhibitor [28]. PCR inhibitors are a very heterogeneous group of chemicals (mostly organic compounds) with different modes of action [29] often considered to be solely amplification inhibition, but inhibition of detection by quenching fluorescence signals has also been reported [30]. Severe inhibition can lead to the loss of alleles from the larger STR loci or to complete false-negative results, which may be erroneously attributed to template degradation. Mild to moderate inhibition may result in a minor loss of alleles and an incorrect estimate of the amount of DNA in the affected sample [28].

Previous studies have shown that the amount of DNA transferred from its original location (primary substrate) to another (secondary substrate) upon physical contact is largely determined by the porosity (surface properties) of both substrates, the state of the DNA-containing medium (dry or wet) and the intensity of contact (“pressure with or without friction” and “passive”) [4, 31]. In the present study, we investigated the influence of solvent-based adhesive removal on secondary DNA transfer under varying test conditions i.e. duration of treatment (up to 5 min) and different initial locations of applied DNA traces (non-adhesive versus adhesive side of the stamp versus envelope). Furthermore, to better assess the extent of downstream effects and relevance to subsequent DNA recovery, mock evidence (DNA-treated stamps affixed to an envelope) was prepared and subjected to dactyloscopic examination — with and without an adhesive removal step.

Human touch depositions are known for inter-replicate and inter-subject variability [5], which poses a significant hurdle to the evaluation of treatment effects on “fingerprint DNA”. Therefore, many previous studies focused on blood or saliva rather than fingerprints. To reduce complexity and variability, we restricted ourselves to the use of extracted DNA in this study. After all, extracellular DNA (from apoptotic epithelial cells, sebaceous glands and sweat glands) can contribute significantly to the DNA content of skin contact samples [5].

## Materials and methods

### Adhesive removal agents

Table 1 contains the list of adhesive removers (organic solvents S1 to S9) used in this study. Included were three groups of chemicals: (i) some commonly used as adhesive removers in forensic dactyloscopic facilities, (ii) others additionally tested and/or used in the dactyloscopic reference laboratory (Criminal Intelligence Service Austria) and (iii) consumer products for industry and private households.

**Table 1:** List of adhesive removers (organic solvents S1 to S9) used in this study

Study code	Name (source information)/ingredients^a^	Boiling point range (°C)^b^	Water solubility^c^	Group
S1	Un-Du® (Un-Du® Products, Inc., St. Louis Park, MN, USA)/naphtha, hydrotreated light	97.8	n.d.	i
S2	Full-Service S400 (Theo Förch GmbH & Co.KG, Neuenstadt, Germany)/aliphatic hydrocarbons (C9–C10), n-alkanes, isoalkanes, cycloalkanes and < 2% aromatics.	n.d.	Insoluble	iii
S3	Un-Stick UNSTK100 Tape Release Agent (Sirchie, Youngsville, NC, USA)/naphtha, hydrotreated heavy (85–90%), (+)-limonene (5–15)	n.d.	Insoluble	i
S4	“BION 1” is an equal mixture (1:1) of petroleum ether (PE) 60–80 °C and PE 80–100 °C; components from Merck KGaA, Darmstadt, Germany	60–100	Practically insoluble resp. insoluble at 25 °C	ii
S5	Toluene (Carl Roth GmbH + Co. KG, Karlsruhe, Germany)	110.6	573 mg/L at 25 °C	i
S6	“Turkish solution” is a mixture (1:2) of cyclohexane and isopropanol; components from Merck	80.7 resp. 82.4	52 g/L at 23.5 °C — partly soluble resp. soluble at 20 °C	i
S7	Petroleum ether 40–60 °C (Merck)	40–60	0.01 g/L at 20 °C	i
S8	“Petrol mixture” is a mixture of acetic acid-n-butyl ester (5%) and petrol 135–180 °C (95%); components from Roth	126 resp. 135–180	5.3 g/L at 20 °C resp. practically insoluble	ii
S9	WD-40 (WD-40 Company, San Diego, CA, USA)/aliphatic hydrocarbons, (C9–C11) (60–80%), n-alkanes, isoalkanes, cycloalkanes and < 2% aromatics, carbon dioxide (1–5%).	176 (active ingredients)	insoluble	iii

^a, b, c^Information on chemical composition, boiling points (at 1.013 hPa) and water solubility was obtained from the manufacturers’ safety data sheets. Based on these data (where available), selected solvents contain volatile organic compounds (VOCs) i.e. organic chemical compounds whose composition allows them to evaporate under normal indoor atmospheric conditions of temperature and pressure

### Effect assessment of solvents on PCR

#### “PCR inhibitor screening” by conventional endpoint PCR

Inhibitor screening was performed by conventional single-plex PCR using a rather “basic” reaction mix [1 × GeneAmp™ PCR buffer (Thermo Fisher Scientific, Darmstadt, Germany), 3 mM magnesium chloride (Thermo Fisher Scientific), 0.25 mM each dNTP (New England Biolabs, Frankfurt, Germany), 2.5 units AmpliTaq Gold™ polymerase (Thermo Fisher Scientific) and primers (F16450, R52 [32], 0.5 μM each, that target a 172 bp portion of the human mitochondrial control region] to amplify human template DNA [10 ng of diluted control DNA from the Quantifiler™ HP (Human Plus) DNA Quantification Kit (Thermo Fisher Scientific)] in a total reaction volume of 25 μL. For inhibition screening, solvents (S1 to S9) were substituted for the water component of the PCR mix (0, 2.5, 5, 10 and 15 μL, respectively) to achieve total concentrations of 0, 10, 20, 40 and 60% (v/v), respectively. The following amplification protocol was applied using the Veriti™ 96-well thermal cycler (Thermo Fisher Scientific): 3 min hot start at 95 °C, followed by 30 cycles at 95 °C (15 s), 58 °C (15 s) and 72 °C (30 s). PCR products were separated by agarose gel (1.5%) electrophoresis in 1 × Tris-borate-EDTA (TBE) buffer.

#### Effect assessment on quantitative real-time PCR

All quantification runs were performed using the Quantifiler HP Kit, MicroAmp™ Optical 96-Well Reaction Plates with MicroAmp Optical Adhesive Film (Thermo Fisher Scientific), the Applied Biosystems™ 7500 Real-Time PCR System (Thermo Fisher Scientific) and the HID Real-Time PCR Analysis Software (version 1.3.1), according to the manufacturer’s instructions [33].

The Quantifiler HP Kit is widely used in forensics for quantitative and qualitative characterization of human nuclear DNA in casework samples. It includes two multicopy genomic targets [small autosomal (SA) and large autosomal (LA), with amplicon sizes of 80 bp and 214 bp, respectively] plus an internal PCR control (IPC) of 130 bp that are amplified simultaneously in the same reaction [33]. The IPC system employs PCR primers, a hydrolysis probe and a synthetic DNA template, to generate a consistent threshold cycle (CT) value in normal samples unaffected by PCR inhibition. An elevated IPC *C*_*T*_ value (compared to quantitation standards) is indicative of the presence of an inhibitor or a high concentration of human template DNA. Thus, the IPC distinguishes between a sample that does not contain human DNA and reactions compromised by the presence of PCR inhibitors, adverse assay setup or instrument failure.

- Confirmation of results from “PCR inhibitor screening”

The following samples were analysed: (a) positive control (1 ng control DNA from the Quantifiler HP kit diluted with THP buffer (provided with the kit)), (b) no template control (amplification-grade water, Promega, Mannheim, Germany) and (c) organic solvents S1 to S9, respectively. All samples (2 μL each; i.e. 10% (v/v) final concentration) were tested in duplicate. The presence of PCR inhibitors was assessed using the internal PCR control (IPC) included in the Quantifiler HP kit.

b) Assessment of target-specific inhibitory effects
  Inhibited test samples were prepared with a fixed level of 0.1 ng/μL of AmpFℓSTR™ DNA Control 007 (Thermo Fisher Scientific) and a range of concentrations (shown in Table 2) of PCR inhibitors S400 (S2), “Turkish solution” (S6) and WD-40 (S9), respectively. A control sample (CTRL) containing the same amount of DNA with no PCR inhibitor was also included. DNA quantitation was performed using the Quantifiler HP Kit and the 7500 Real-Time PCR System. Inhibitory effects were monitored using mean quantitation results for each assay target [small autosomal target (SA), long autosomal target (LA) and internal PCR control (IPC)] of the Quantifiler HP Kit.

**Table 2:** Results from Quantifiler HP Kit analysis of inhibited test samples

Sample	Inhib. conc.^a^ (% (v/v))	Quantifiler HP Kit results^b^			
		SA^c^	LA^c^	IPC *C*_*T*_^d^	DI
CTRL	0	1.13 ± 0.11	1.13 ± 0.17	27.80 ± 0.21	-
S400 (S2)	0.8	1.19 ± 0.01	1.12 ± 0.02	27.80 ± 0.09	1.07 ± 0.01
	1.0	2.26 ± 0.96	0.97 ± 0.34	27.87 ± 0.05	2.91 ± 2.03
	2.0	Undet.	Undet.	Undet.	-
	4.0	Undet.	Undet.	Undet.	-
	6.0	Undet.	Undet.	Undet.	-
	8.0	Undet.	Undet.	Undet.	-
	10.0	Undet.	Undet.	Undet.	-
Turkish solution (S6)	1.0	1.21 ± 0.11	1.03 ± 0.34	27.92 ± 0.05	1.23 ± 0.30
	2.0	1.61 ± 0.16	1.06 ± 0.27	28.02 ± 0.07	1.59 ± 0.55
	4.0	3.06 ± 0.08	0.73 ± 0.30	27.97 ± 0.03	4.58 ± 1.96
	6.0	Undet.	Undet.	Undet.	-
	8.0	Undet.	Undet.	Undet.	-
	10.0	Undet.	Undet.	Undet.	-
WD-40 (S9)	1.0	1.08 ± 0.02	1.40 ± 0.14	27.92 ± 0.01	1.30 ± 0.02
	2.0	3.08 ± 0.21	0.71 ± 0.13	28.20 ± 0.02	4.46 ± 1.13
	4.0	2.42 ± 1.20	0.35 ± 0.01	28.13 ± 0.10	6.86 ± 3.51
	6.0	8.33 ± 7.40	0.04 ± 0.00^**c**^	27.98 ± 0.14	70.28 ± 0.00 ^**e**^
	8.0	Undet.	Undet.	Undet.	-
	10.0	Undet.	Undet.	Undet.	-

Inhibited test samples were prepared with a fixed level of 0.1 ng/μL of human male DNA (007) and a range of concentrations of PCR inhibitors [S400 (S2), “Turkish solution” (S6) and WD-40 (S9), respectively]. A control sample (CTRL) containing the same amount of DNA with no PCR inhibitor was also included. Real-time PCR analysis of samples was performed using the Quantifiler HP Kit, the 7500 Real-Time PCR System and the HID Real-Time PCR Analysis Software, according to the manufacturer’s specifications. The table shows mean quantitation results for each assay target [small autosomal target (SA), long autosomal target (LA) and internal PCR control (IPC)]. Degradation index ((DI) i.e. the ratio of quantities between autosomal target amplicons of different fragment sizes: small (80 bp) versus large target (214 bp)) was automatically calculated by the analysis software

^a^Total concentration of inhibitor in the reaction

^b^Quantification results for assay targets (mean of duplicate assays)

^c^Measured mean concentration of target [ng/μL]

^d^Mean threshold cycle (*C*_*T*_) values of the IPC

^e^Due to amplification failure only one out of two LA assays was detected in the presence of 6% (v/v) WD-40

#### Effect assessment on STR profiling

To examine the inhibitory effects of selected adhesive removers on short tandem repeat (STR) profiling we used the Applied Biosystems™ NGM Detect™ PCR Amplification Kit (Thermo Fisher Scientific) [34]. The amplification performance of the “Integrated Quality Control System” (IQC) of the kit was used to evaluate the success of the PCR reaction, provide an indication of sample quality and distinguish samples that are degraded from those that contain PCR inhibitors. Male human genomic DNA (2800M Control DNA, Promega, 0.5 ng each) was subjected to PCR in a total reaction volume of 25 μL using the Veriti thermal cycler applying amplification conditions recommended by the manufacturer. For inhibition screening, chemical solvents (S2, S6 and S9, respectively) were substituted for the water component of the PCR mix (0, 0.5, 1.25, 2.5, 5 and 7.5 μL, respectively) to achieve total concentrations of 0, 2, 5, 10, 20 and 30% (v/v), respectively. PCR products were analysed on the Applied Biosystems™ 3500 Genetic Analyzer (Thermo Fisher Scientific) using GeneMapper ID-X software v. 1.6.

### Effect assessment of solvents on DNA recovery

#### Effects of “dry exposure” on DNA recovery

Solvent-DNA master mixes were prepared shortly before the start of the incubation time series by adding an aliquot of control DNA from the Quantifiler HP Kit (diluted in THP buffer) to an organic solvent (S1 to S9, respectively) in a microcentrifuge tube (“low retention”; Kisker Biotech). An aqueous DNA solution using water (amplification-grade, Promega) instead of organic solvent was prepared and served as a control. Each sample was mixed by pipetting and aliquots of about 50 μL (each containing 50 ng DNA) were pipetted to the cotton tips of sterile swabs (Voigtlaender Polizei- und Kriminaltechnik, Blumberg, Germany). The swabs (eight swabs for each type of DNA-solvent mixture) were mounted on a tube stand with the wooden handle fixed and the cotton tip “free” so that the solvent-DNA mixture could evaporate and dry freely. A time series experiment was conducted including the following time points of incubation: 5 min, 1 h, overnight (18 h) and 1 week, respectively. Each combination (type of sample/solvent and time point) was tested in duplicate. The incubation was carried out at room temperature and in the dark. The LOG32 TH portable data logger (Airflow Developments Limited, High Wycombe, UK) was used to monitor storage conditions and recorded temperature (min/max/average: 21.7/29.6/22.4 °C) and humidity (min/max/avg: 34.1/72.3/61.9% rH). Immediately after the indicated incubation period, DNA was isolated from swabs using the QIAamp® DNA Mini Kit (Qiagen, Hilden, Germany) with a final elution volume of 50 μL. Extraction blanks (clean swabs) served as negative controls and were carried through the entire process of DNA purification and quantitation. Quantitation of the recovered human nuclear DNA was carried out in duplicate using the Quantifiler HP Kit and the 7500 Real-Time PCR System, according to the manufacturer’s specifications [33]. Degradation index ((DI) i.e. the ratio of quantities between autosomal target amplicons of different fragment sizes: small (80 bp) versus large target (214 bp)) was automatically calculated by the analysis software. The presence of PCR inhibitors was assessed by comparing threshold cycle (CT) values of the internal PCR control (IPC) between samples and controls. For a graphical interpretation of quantitation results, the numerical data from real-time PCR were exported to Microsoft Excel 2016 (Microsoft Corporation, Redmond, USA) software and used to calculate descriptive statistical values (arithmetic mean and standard deviation) for corresponding samples. Bar charts were created by plotting the quantities (calculated mean values for the small autosomal target, degradation index (DI) and internal PCR control (IPC), respectively; on the *y*-axis) against the incubation time points (on the *x*-axis; in ordinal scale).

### Effects of “wet exposure” on DNA recovery

The procedure of the “wet exposure” study was similar to that of the “dry exposure” study ( “Effects of “dry exposure” on DNA recovery”), except that the samples were not incubated on swabs (“free to air”) but in closed sample tubes with screw cap (2 mL; included in the EZ1 DNA Tissue Kit, Qiagen) to reduce evaporation of volatile organic compounds. Solvent-DNA master mixes were prepared shortly before the start of the incubation time series by adding an aliquot of control DNA from the Quantifiler HP Kit (diluted in THP buffer) to an organic solvent (S1 to S9, respectively) in a microcentrifuge tube (“low retention”; Kisker Biotech, Steinfurt, Germany). An aqueous DNA solution using water (amplification-grade, Promega) instead of organic solvent was prepared and served as a control. Each sample was mixed by repetitive pipetting up and down and aliquots of about 100 μL (each containing 50 ng DNA) were transferred to screw-capped sample tubes (Qiagen) (eight tubes for each type of DNA-solvent mixture). Immediately afterwards, the screw cap was closed tightly, wrapped with parafilm (Sigma-Aldrich, Taufkirchen, Germany) and the incubation started. A time series experiment was conducted including the following time points of incubation: 5 min, 1 h, overnight (18 h) and 1 week, respectively. The incubation was carried out at room temperature and in the dark. Each combination (type of sample/solvent and time point) was tested in duplicate. DNA was isolated using the QIAamp kit with a final elution volume of 50 μL. Analysis of recovered DNA by real-time PCR and the graphical evaluation of results using MS Excel (2016) was conducted without deviations from the procedure described in “Effects of “dry exposure” on DNA recovery”.

### Effect assessment of solvent-based adhesive removal processes on the subsequent analysis of fingerprint and DNA

A series of adhesive-removal experiments was conducted with prepared “mock evidence” (self-adhesive postage stamps on paper envelope with deposition of either fingermark or DNA) to investigate the impact of treatment time and location of applied traces on fingerprint detection and DNA analysis, respectively.

#### Effects of adhesive removal processes on the subsequent visualisation of latent fingerprints

Self-adhesive postage stamps of the type “Salzburg, Loewenpranke € 0.25” (25 × 25 mm^2^, Austrian postal service, product number: 0117811) and white paper envelopes self-seal white paper envelopes (format C5, 100 g/m^2^, Elco, Switzerland, product number: 74535.12) were used. Fingermarks of comparable quality were applied to the adhesive side of the stamps. The stamps were then affixed to the paper envelopes and stored for a week at an office desk in moderate sunlight and at an average room temperature of 24 °C before further processing. In order to create conditions that closely resemble procedures used in routine forensic dactyloscopy, the prepared specimens (fingerprint-marked stamps affixed to envelope) were first subjected to ninhydrin treatment (see below) to visualise latent fingerprints on porous substrates (i.e. the envelope). Afterwards, adhesive removal (i.e. the separation of affixed stamps from the envelope) was conducted with the aid of the following organic solvents: Un-Du (S1), petroleum ether (PE) (boiling point: 60–80 °C), petroleum ether (80–100 °C), BION1 (S4) (1:1 mixture of PE 60–80 °C and PE 80:100 °C) and “Turkish solution” (S6), respectively. A time-series experiment of solvent-treatment prior to the physical separation of the stamp from the envelope was performed and included the following time points: 5, 10, 15 and 25 s, respectively. Notably, the solvents were not applied directly to the stamp, but to the envelope (opposite the affixed stamp). In order to assess the effects of adhesive removal procedures on dactyloscopy, the adhesive side of the stamps was subjected to treatment with carbon black powder (see below) to visualise latent fingerprints.

- Ninhydrin treatment
  The method is widely used for the visualisation of latent fingerprints (or bloody marks) on porous or semi-porous surfaces [24]. For this purpose, a working solution [ninhydrin (3 g) dissolved in ethanol (20 mL) and filled up with petroleum ether (PE 40–60 °C) to a total volume of 0.5 L] was prepared. Samples were briefly soaked in the reagent, air dried and placed in a climate chamber (NINcha S31 from Attestor Forensics, Bad Wurzach, Germany) for 48 h at 35 °C and 65% relative humidity for development. The amino acids present in the fingerprint react with the ninhydrin solution and stain the trace reddish-purple reaching a maximum intensity within 48 h.

b) Carbon black powder
  The method was used for the visualisation of latent marks on adhesive surfaces. For this purpose, an adhesive suspension [ether sulfate 70% (15 g) dissolved in 85 mL water and mixed with carbon black powder (10 g); stirred before use] was prepared and applied to the adhesive surface (brush/pipette). Resultant marks become visible and black in colour.

#### Effect assessment of adhesive removal processes on DNA recovery from prepared specimens (postage stamps attached to envelope)

Self-adhesive stamps of the type “Fiaker-Melone-Wien” (25 × 25 mm^2^, Austrian postal service, product number: 1107814) and self-seal white paper envelopes (format C5, 100 g/m^2^, Elco, Switzerland, product number: 74535.12) were used. Aliquots of 5 μL of human nuclear DNA solution (with 50 ng of control DNA from the Quantifiler HP DNA Quantification Kit diluted in THP buffer) were applied to three different locations: (a) the adhesive side of a stamp, (b) the non-adhesive side of a stamp and (c) on the envelope (in the centre of drawn square areas of 25 × 25 mm^2^). DNA traces applied to the adhesive side were air dried for 2 h at room temperature before the stamps were affixed to the envelope. Stamps (type b, with DNA on the non-adhesive side) were affixed to the envelope prior to the application of DNA. After deposition of DNA to the marked areas, the envelope (type c) was allowed to dry for 2 h at room temperature before stamps (without DNA) were affixed to these marked areas. All envelopes (specimens type a–c) were stored overnight (18 h) at room temperature and in the dark. The LOG32 TH portable data logger (Airflow Developments) was used to record temperature (min/max/avg: 22.2/23.7/22.5 °C) and humidity (min/max/avg: 45.0/58.1/50.5% rH).

Before adhesive removal treatment, all the prepared stamps were cut out of the envelopes, but leaving the adherent paper stuck to the back. One corner of each stamp was detached from the paper with tweezers in advance to allow for quick and smooth separation later. Using tweezers, each stamp was held horizontally with the back upwards and 300 μL of “Turkish solution” (S6) was pipetted to its centre. A time-series experiment of solvent-treatment prior to adhesive release was conducted using an additional pair of tweezers (for separation) and included the following time points: 10, 60 and 300 s and untreated control (0 s). Because the complete separation required about 5 s, the detachment process was started correspondingly earlier. Separated stamps and associated pieces of paper were placed on clean glass dishes standing in an upright position to dry (~2 h) at room temperature.

The QIAamp DNA Mini Kit (Qiagen) was utilized for DNA recovery. DNA isolation from stamps included swabbing the DNA-treated side (using a wet cotton tip moistened with buffer ATL and executing combined rolling and rubbing movements for at least 1 min). Thereafter, each stamp was cut to small pieces with a clean blade and transferred to a fresh 2-mL tube. After addition of the associated cotton tip (cut-off) and 400 μL of ATL buffer (Qiagen), the tubes were agitated (1000 rpm) at 56 °C on a thermal mixer for 2 h.

Swab sampling from paper bears the risk of losing sample material by attrition; therefore, no swabbing was performed for DNA purification from paper. Envelope paper was cut to small pieces with a clean blade and transferred to a 2-mL tube. After addition of 400 μL of ATL buffer (Qiagen), the tubes were agitated (1000 rpm) at 56 °C on a thermal mixer for 2 h.

The subsequent procedure was identical for both types of samples. Sample materials (swabs and cut stamp and cut envelope paper, respectively) were transferred to a spin filter unit using a clean toothpick. The filter unit (containing the materials) was placed back into the original 2-mL receiver tube, and the entire assembly was subjected to centrifugation (20,000 × g for 5 min). Thereafter, filter elements were discarded, and 400 μL of lysis buffer AL (Qiagen) was added to each remaining sample (i.e. filtrate). Samples were subjected to vortexing and incubation on a thermomixer for 10 min at 70 °C. Then 400 μL of absolute ethanol was added, and samples were transferred to QIAamp Spin Columns (placed in a 2-mL collection tubes) and centrifuged at 6000 × g for 1 min. The subsequent washing steps were carried out according to the manual of the kit (see also “Effects of “dry exposure” on DNA recovery”), and DNA was eluted in 50 μL of elution buffer AE (Qiagen). Quantitation of the recovered human nuclear DNA was carried out in duplicate using the Quantifiler HP Kit, the 7500 Real-Time PCR System and the HID Real-Time PCR Analysis Software according to the manufacturer’s specifications. For a graphical interpretation of quantitation results, the numerical data from real-time PCR were exported to MS Excel (2016) and used to calculate descriptive statistical values (arithmetic mean and standard deviation) for the corresponding samples.

#### Effect assessment of combined adhesive removal and fingerprint development processes on subsequent DNA recovery from mock evidence (postage stamps attached to envelope)

In order to better estimate the extent of the downstream effects of adhesive removal processes, compared to conventional fingerprint development processes on subsequent DNA recovery, mock evidence (DNA-treated stamps affixed to an envelope) was prepared and subjected to dactyloscopic examination — with and without an adhesive release step.

For this purpose, four test samples (envelopes with DNA deposition, E1 to E4) were prepared. Nine square areas (25 × 25 mm^2^) were drawn (in three rows of three squares each) on each envelope (self-seal white paper, format C5 and 100 g/m^2^, Elco, Switzerland) and treated (from left to right) with DNA (50 ng of control DNA from the Quantifiler HP Kit, diluted in THP buffer), fingermark and untreated (control), respectively. In addition, nine self-adhesive postage stamps (type “Fiaker-Melone-Wien” (25 × 25 mm^2^, Austrian postal service)) were affixed to each envelope in three rows of three. The upper row of stamps served as a control (no DNA). In the second row, DNA (50 ng control DNA each) was applied to the non-adhesive side of the stamps. In the third row, the stamps were first treated with DNA (50 ng control DNA each) on the adhesive side and later affixed to the envelope. DNA traces applied to the adhesive side were allowed to dry for 2 h at room temperature before the stamps were affixed to the envelope. Prepared test specimens were stored at room temperature in the dark for 1 week. Storage conditions monitoring with the LOG32 TH portable data logger (Airflow Development) recorded temperature (min/max/avg: 22.4/27.1/23.2 °C) and humidity (min/max/avg: 44.2/55.7/52.5% rH).

- Overview of treatments (test envelopes E1 to E4)
  Three envelopes (E1 to E3) were processed first using 1,2-indanedione-zinc (Ind-Zn), followed by ninhydrin treatment and incubation in a climate chamber for 48 h. Then, the envelopes were cut open laterally. Removal of the stamps was performed either with the aid of adhesive removers (E1: “Turkish solution”; E2: Un-Du) or purely mechanically using tweezers (E3). Envelope E4 was left untreated (no Ind-Zn/ninhydrin), and stamps were removed mechanically. DNA was isolated from stamps and paper using the QIAamp DNA Mini Kit (details of the procedure are provided in “Effect assessment of adhesive removal processes on DNA recovery from prepared specimens (postage stamps attached to envelope)”). Quantitation of human nuclear DNA was carried out in duplicate using the Quantifiler HP Kit, the 7500 Real-Time PCR System and the HID Real-Time PCR Analysis Software, according to the manufacturer’s specifications.

b) 1,2-Indanedione-zinc (Ind-Zn) treatment
  The 1,2-indanedione-zinc method is commonly applied to visualise latent dactyloscopic traces on porous surfaces and used before treatment with ninhydrin [24]. For this purpose, a working solution was prepared by dissolving 1.0 g of indanedione in 90 mL ethyl acetate and the sequential addition of acetic acid (10 mL), petroleum ether 40–60 °C (900 mL) and 10 mL of zinc solution (0.667% (w/v) of zinc chloride in ethanol). Envelopes were treated twice with the Ind-Zn working solution. Traces were developed for 10 s at 160 °C in the transfer press (Secabo TC7). Ind-Zn reacts with amino acids to form a strongly fluorescent dye under suitable excitation conditions.

DNA was isolated from stamps and paper using the QIAamp DNA Mini Kit (details of the procedure provided in “Effect assessment of adhesive removal processes on DNA recovery from prepared specimens (postage stamps attached to envelope)”). Quantitation of human nuclear DNA was carried out in duplicate using the Quantifiler HP Kit, according to the manufacturer’s specifications. For a graphical interpretation of quantitation results, the numerical data from real-time PCR were exported to MS Excel (2016) to calculate descriptive statistical values (arithmetic mean and standard deviation) for the corresponding samples.

### Data analysis

Statistical calculations were performed using MS Excel (2016) with the Real Statistics Resource Pack software add-in (Release 7.6) [35]. Quantification data (small autosomal target, degradation index (DI) and IPC C_T_, respectively) obtained from real-time PCR analysis with the Quantifiler HP Kit were assigned to treatment groups and tested for normality applying a Shapiro-Wilk test. To determine if the data sets were significantly different from each other, either a one-tailed *t*-test for independent samples (assuming normal distribution) was applied (significance level *p* < 0.05). Analysis of variance (ANOVA) was used to compare the means among three or more independent treatment groups. ANOVA with repeated measures was employed to assess the impact of the same treatment at different time intervals. The Tukey’s Honest Significant Difference test (Tukey HSD test) with Bonferroni correction served as post hoc test for pairwise comparisons between groups.

## Results and discussion

In the present study, we investigated the effects of solvent-based adhesive removal processes on the subsequent detection and analysis of fingerprint and DNA, focusing on PCR inhibition, DNA transfer and recovery.

### Effect assessment of solvents on PCR

The potential of adhesive removers (solvents S1 to S9) to exert inhibitory effects on PCR was determined in three steps. First, identification of inhibitory compounds (“PCR inhibitor screening”). Second, confirmation and (semi-)quantitative evaluation of the observed inhibitory effects by multiplex real-time PCR using the Quantifiler™ HP Kit (Thermo Fisher Scientific), and third, the generation of STR profiles of inhibited samples using the NGM Detect™ Kit (Thermo Fisher Scientific), developed for the analysis of challenging casework samples.

**Fig. 1:**
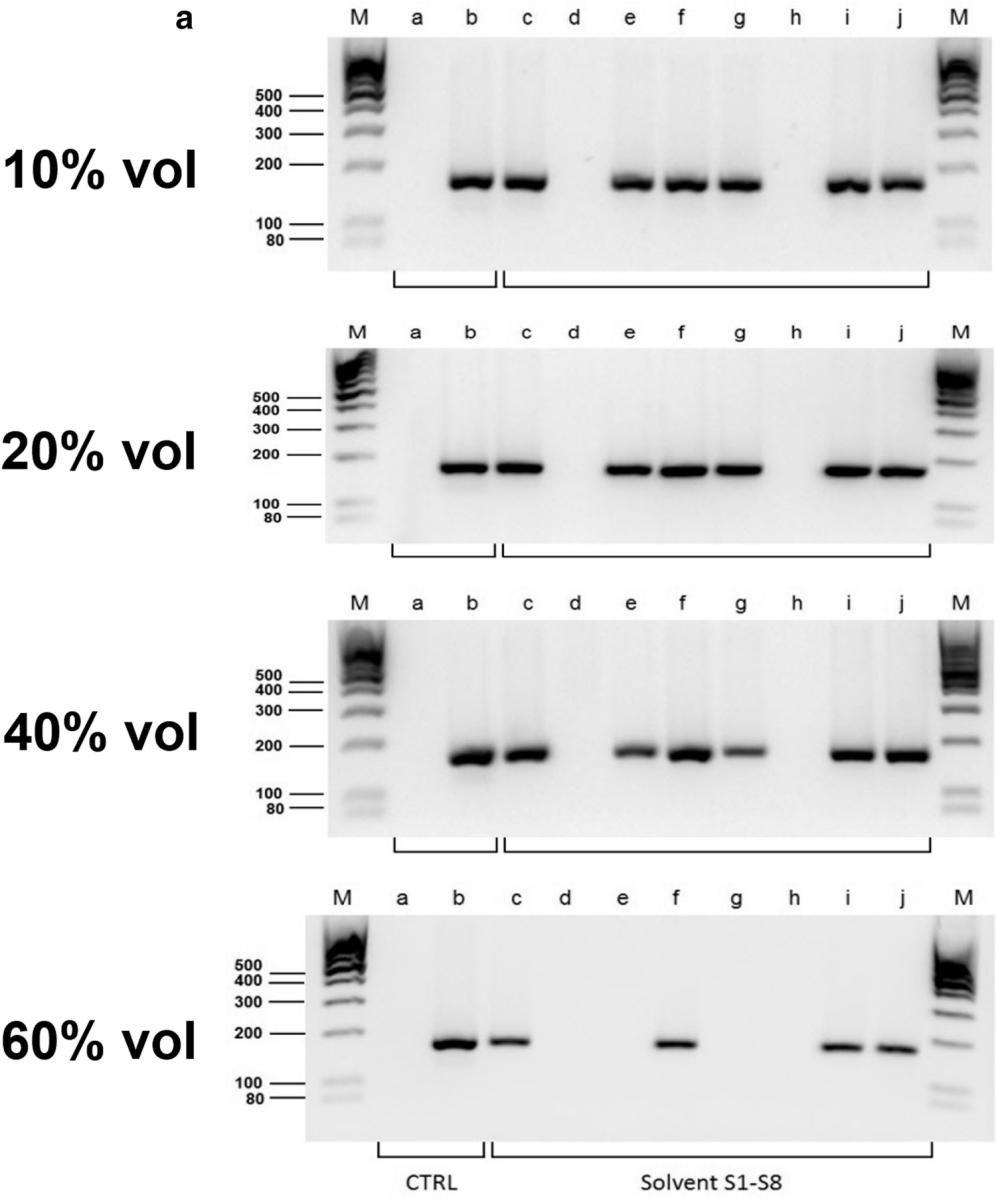

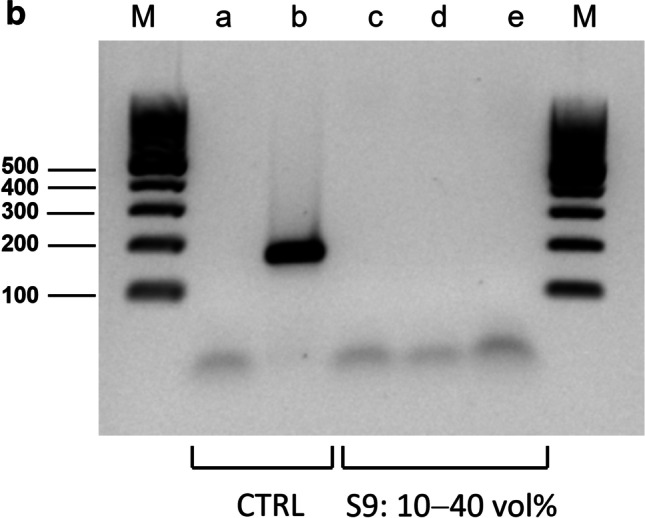
**a** **“Inhibitor screening” showed strong PCR inhibition with solvents S400 and “Turkish solution”**. PCR products generated with template DNA (10 ng each), primers that target a 172 bp fragment of human mitochondrial DNA (mtDNA) and in the presence of various amounts of chemical solvents (S1–S8) were separated by agarose gel (1.5%) electrophoresis in 1 **×** TBE buffer. Aliquots of 5 μL from the PCRs were loaded. Lanes M: DNA ladder (low range, Thermo Fisher Scientific), lanes a–b: controls (CTRL) (lane a: no template control, lane b: amplification control (no solvent added)), lanes c–j: PCR was conducted in the presence of either of the following solvents; c: Un-du (code: S1), d: S400 (S2), e: Un-Stick (S3), f: BION1 (S4), g: Toluene (S5), h: “Turkish solution“ (S6), i: petroleum ether (7), and j: “Petrol mixture” (S8). The solvents had been substituted for the water component of the PCR mix to achieve a total concentration of 10% (first panel), 20% (second panel), 40% (third panel) and 60% (v/v) (fourth panel), respectively. The results indicated a strong inhibition potential for the solvents S400 and “Turkish solution”, and a relatively weak inhibitory effect with Un-stick and Toluene. No PCR inhibition was observed with the other solvents. **b** **“Inhibitor screening” showed strong PCR inhibition with WD-40 (S9)**. PCR products generated with template DNA (10 ng each), primers that target a 172 bp fragment of human mtDNA and in the presence of various amounts of WD-40 (solvent S9) were separated by agarose gel (1.5%) electrophoresis in 1 **×** TBE buffer. Aliquots of 5 μL from PCRs were loaded. Lanes M: DNA ladder (GeneRuler 100 bp, Thermo Fisher Scientific), lanes a–b: controls (CTRL) (lane a: no template control, lane b: amplification control (without addition of solvent)), lanes c–e: PCR was conducted in the presence of WD-40 in a total concentration of 10% (lane c), 20% (d) and 40% (v/v) (e), respectively

“Inhibitor screening” was performed with endpoint PCR using the AmpliTaq Gold® DNA polymerase (a chemically modified hot-start DNA polymerase without particular inhibitor tolerance [36]), a very rudimentary, simple reaction setup without additives (e.g. PCR facilitators) and primers targeting a relatively short fragment (172 bp) of (high-copy) mitochondrial DNA i.e. overall conditions that allowed efficient amplification but were sensitive to interference. Because inhibition is dose-dependent [28, 29], the solvents (S1 to S9) were tested in different concentrations (from 10 to 60% by volume). Analysis by agarose gel electrophoresis (Fig. 1a, b) identified three potent inhibitors (S400, “Turkish solution”, WD-40) that caused complete failure of amplification at all test concentrations, two solvents (Un-Stick, toluene) with a weak inhibitory potential observed only at the highest concentrations, and four solvents (Un-Du, BION1, petroleum ether, “petrol mixture”) that caused no inhibition under the given conditions.

Next, we used the internal PCR control (IPC) of the Quantifiler HP Kit as a molecular tool to evaluate the inhibitory potential of each solvent (S1 to S9) at a final concentration of 10% by volume and without addition of human template DNA (Supplementary Fig. 1). Multicomponent view of the IPC raw data (reporter fluorescence data plotted against cycle number) displayed a flat region corresponding to the baseline, followed by a rapid increase in fluorescence with the onset of exponential amplification in controls (NTC and POS) and presumably un-inhibited samples (e.g. Un-Du), but not in assays containing the suspected inhibitors (S400, “Turkish solution” and WD-40), where no significant increase in fluorescence was observed. Amplification of the IPC seemed grossly inhibited by these three solvents, but not by the others, confirming the initial “screening” results.

Inhibitor assessment generally depends on the assumption that inhibition affects all PCR reactions to the same extent. However, differential susceptibility to PCR inhibition across different assays has been reported [37], thus the effects of inhibitors on the IPC are not necessarily predictive of the effects on other targets [38]. In order to examine potential target-specific differences, inhibited test samples were prepared to contain a fixed amount of 0.1 ng/μL of DNA Control 007 and a range of concentrations of PCR inhibitors S400, “Turkish solution” and WD-40, respectively (Table 2). Inhibitor concentrations covered a range from no PCR inhibition to complete failure of amplification. Complete failure of amplification (all targets) was observed at 2% of S400, 6% of “Turkish solvent” and 8% (v/v) of WD-40, respectively (Table 2). Interestingly, the IPC was the most robust target and provided consistent quantification results at lower inhibitor concentrations (before complete amplification failure), whereas the autosomal quantification targets (SA and LA) showed pronounced, but opposite effects. As expected from the longer amplicon, the LA target was most sensitive to inhibition, since its amplification significantly decreased as a function of inhibitor concentration. In contrast, amplification of the SA target was progressively enhanced until a sudden drop to complete inhibition. Consequently, the SA:LA ratio (the “Degradation Index”) increased with elevated inhibitor concentrations.

**Fig. 2:**
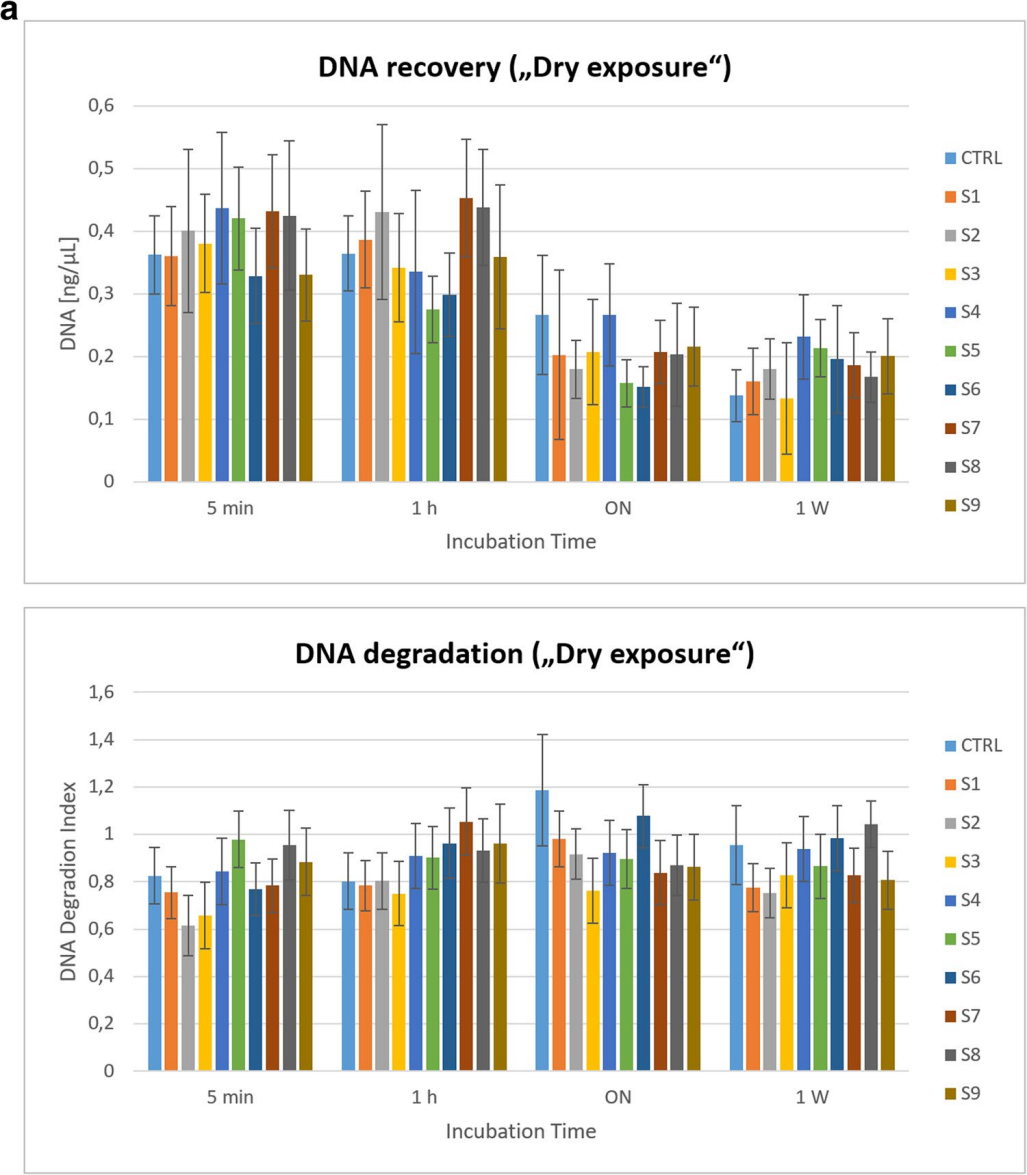

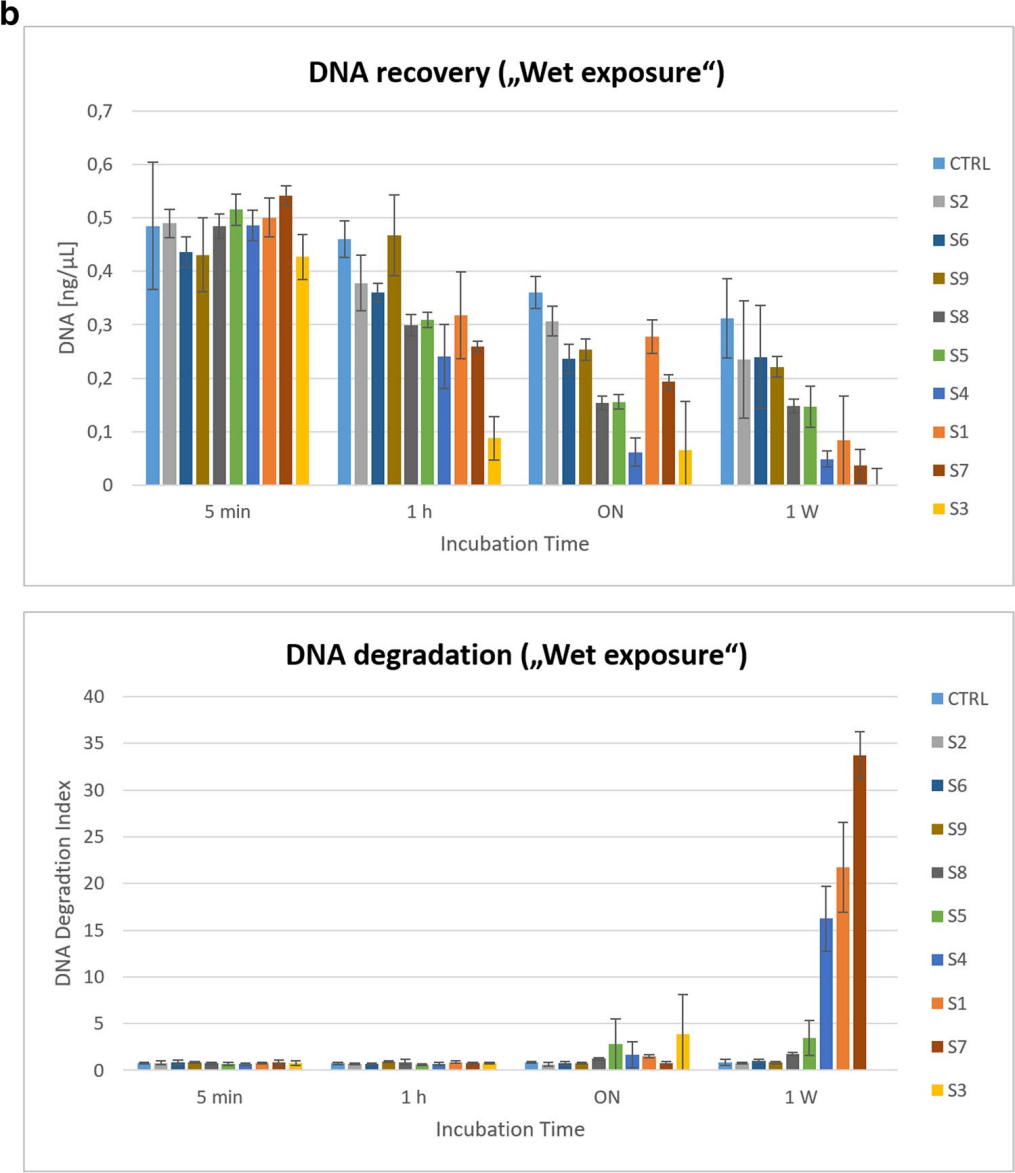
**a** **“Dry exposure” assessment revealed no enhanced adverse effects of solvents (S1–S9) on DNA quantity and quality, compared to water**. The bar graphs shown were generated using MS Excel (2016) by plotting DNA quantitation results from real-time PCR analysis [calculated mean values for the small autosomal target (DNA [ng/µL], upper panel) and degradation index (DI) (lower panel)], respectively; each on the *y*-axis)] against the incubation time (on the *x*-axis; in ordinal scale). Mixed samples (50 μL aliquots of solvents S1 to S9 and water as control (CTRL), respectively, each spiked with 50 ng DNA) were applied to sterile cotton swabs. Swabs were incubated at room temperature (RT) in the dark for five minutes (5 min), one hour (1 h), 18 h (overnight, ON) or one week (1 w), allowing the solvent-DNA mixture to evaporate and dry freely. Each combination (type of solvent and time point) was tested in duplicate samples. Subsequently DNA was purified from swabs and subjected to quantitation using the Quantifiler HP Kit. **b** **“Wet exposure” assessment revealed pronounced adverse effects of certain solvents (S3 > S7 > S1 > S4) on the quantity and quality of recovered DNA**. The bar graphs shown were generated using MS Excel (2016) by plotting DNA quantitation results from real-time PCR analysis [calculated mean values for the small autosomal target (DNA [ng/µL], upper panel) and degradation index (DI) (lower panel), respectively; each on the *y*-axis)] against the incubation time (on the *x*-axis; in ordinal scale). Mixed samples (100 μL aliquots of solvents S1 to S9 and water as control (CTRL), respectively, each spiked with 50 ng DNA) were transferred to closed sample tubes to prevent the evaporation of volatile organic compounds and incubated at room temperature (RT) in the dark for five minutes (5 min), one hour (1 h), 18 h (overnight, ON) or one week (1 w). Each combination (type of solvent and time point) was tested in duplicate samples. Subsequently DNA was purified and subjected to quantitation using the Quantifiler HP Kit

Since the IPC showed no inhibition, DNA degradation could be a likely cause for the different DNA concentrations in SA and LA. As DNA is degraded, amplification of longer fragments (LA) is expected to decrease, while shorter fragments (SA) are preferentially amplified, which can be observed as a “ski slope” pattern in STR profiles, a phenomenon that was indeed observed at lower solvent concentrations (see below). At the higher concentration, inhibition seems to be the most likely cause. Since the solvent was added directly to the PCR reaction, high temperatures, e.g. during denaturation, could have accelerated DNA degradation. However, no significant increase in DNA degradation was observed upon exposure (dry and wet) to these three solvents (S2, S6 and S9), compared to the aqueous DNA control (Fig. 2a, b). Furthermore, in the case of solvent-induced template depletion, a general decline in amplification would have been expected for both targets (LA and SA, albeit to a lesser extent for the later), which was obviously not the case (Table 2). The data suggest that additional factors (e.g. sequence differences) may also have contributed to the observed effects.

The melting temperature depends on the length of a DNA molecule and its specific nucleotide sequence. By adding a solvent, the DNA’s melting temperature can be lowered [39] until, for example, a solvent concentration is reached at which the actual annealing temperature of the primers in a PCR has changed so much that it is no longer compatible with the original thermal protocol. On the other hand, solvents are widely used as “PCR facilitators”, e.g. in order to reduce the formation of inhibitory secondary structures caused by palindromic motifs in GC-rich sequences [28]. Thus, the addition of a solvent in a multiplex PCR can have opposite effects on the amplification efficiency of different targets, at least in part also depending on the target sequence.

The NGM Detect™ Kit (Thermo Fisher Scientific) was used to assess potential effects of inhibitors [S400 (S2), “Turkish solution” (S6) and WD-40 (S9)] on STR profiling in concentrations that covered a range from no PCR inhibition to complete failure of amplification. Samples were tested using the recommended optimal amount (0.5 ng) of template DNA [34]. Complete failure of amplification (all targets) was observed at 10% (“Turkish solution”), 20% (S400) and 30% (v/v) (WD-40), respectively (Supplementary Fig. 2a–d). The NGM Detect™ belongs to a newer generation of commercial STR amplification kits with improved sensitivity and shortened STR markers, especially designed for the analysis of difficult case samples with poor DNA quality (high degradation index and presence of inhibitors) and low quantity [40]. The quality control system (IQCS and IQCL; synthetic DNA targets included in the primer mix) featured by the kit can be used to distinguish between successful amplification, failed PCR amplification and complications due to the lack of template, DNA degradation and PCR inhibition [34]. The presence of both the IQCS and IQCL with balanced relative peak heights suggests that PCR has occurred optimally, whereas a significantly lower IQCL peak height relative to the IQCS indicates that the PCR reaction has been compromised by inhibition. Accordingly, electropherograms of amplification products obtained with the NGM Detect™ Kit mostly displayed ski slope profiles with decreased IQCL peak height as a function of inhibitor concentration (until complete amplification failure) that in combination are typical indicators of PCR inhibition (Supplementary Fig. 2a–c). Interestingly, assays spiked with “Turkish solution” showed some evidence of target-specific differential effects on amplification (Supplementary Fig. 2d), whereas amplification with the other two solvents (S400 and WD-40) resulted in typical ski slope profiles (data not shown). With increasing concentrations of “Turkish solution”, certain targets were selectively favoured [e.g. TH01 (Supplementary Fig. 2d), D19S433, FGA (data not shown)], while some others (e.g. amelogenin; Supplementary Fig. 2e) were selectively impaired.

### Effect assessment of solvents on DNA recovery

Adhesive removers (solvents S1 to S9) of this study contain volatile organic compounds that vaporize under normal indoor atmospheric conditions of temperature and pressure (Table 1). To investigate possible effects of direct exposure and volatility on DNA stability, mixed samples (DNA and solvent) were prepared and subjected to a series of time-course experiments under conditions where evaporation was unhindered (“dry exposure”) and conditions where evaporation was prevented (“wet exposure”), respectively.

#### Quantitative real-time PCR analysis of DNA recovered from “dry exposure” to organic solvents (S1 to S9)

Aliquots of ~ 50 μL of solvents (S1 to S9, respectively; water as control) supplemented with 50 ng DNA were applied to sterile cotton swabs allowing the volatile compounds to evaporate and dry freely (“dry exposure”). Time series experiments were performed with the following incubation times: 5 min, 1 h, overnight (18 h) and 1 week, respectively. DNA was purified from swabs and subjected to quantitative real-time PCR analysis (Fig. 2a). The controls (aqueous DNA solution) gave similar quantitative results to the test samples (exposure to solvents S1 to S9). Average yields of ~39% (5-min group) were obtained for the initial DNA amounts of 50 ng and only about half after 1 week. Importantly, data from real-time PCR analysis indicated no particular impairment of DNA quality in solvent-treated test samples, compared to aqueous controls (Fig. 2a and Supplementary Table 1a). The SA:LA ratio (i.e. “degradation index” (DI)) showed no evidence of degradation in the vast majority of samples (DI < 1) and reached a maximum of 1.42 (slight degradation). The IPC *C*_*T*_ values of the test samples ranged from 26.97 (minimum) to 28.51 (maximum). Thus, they were not strongly elevated, compared to controls and quantification standards, but minor disturbances cannot be excluded. In summary, with unimpeded evaporation and under the given experimental conditions (duration, temperature and swab as substrate), none of the solvents appeared to have particularly deleterious effects on DNA in direct comparison with water.

#### Quantitative real-time PCR analysis of DNA recovered from “wet exposure” to organic solvents (S1 to S9)

The procedure of the “wet exposure” study was similar to that of the “dry exposure”, except that samples were placed in closed test tubes rather than on swabs (“free in air” to prevent evaporation of volatile organic compounds. Aliquots of 100 μL of solvent (S1 to S9, respectively; water as control) supplemented with 50 ng DNA were incubated at room temperature for 5 min, 1 h, overnight (18 h) and 1 week, respectively. DNA was purified and subjected to quantitative real-time PCR analysis (Fig. 2b and Supplementary Table 1b). The extraction efficiency was significantly increased in the “wet exposure” liquid test samples, compared to the “dry exposure” swab samples (average 48 vs. 39% DNA recovery at 5 min incubation). The average DNA concentration in eluates purified from solvent-treated test samples (~0.13 ng/μL) was significantly reduced after 1 week of incubation, compared to the early time intervals or aqueous controls. After overnight treatment, certain solvent samples (Un-Du, Un-Stick, BION1, Toluene, and “Petrol mixture”) showed initial signs of slight to moderate degradation [degradation index (DI) 1 to 10, IPC *C*_*T*_ flag not triggered]. Severe degradation [DI > 10 or blank (no value), IPC *C*_*T*_ flag not triggered] was observed after 1 week of incubation with the following solvents Un-Du (S1; DI 21.71), Un-Stick (S3; blank; i.e. no DI available due to deficient LA target amplification), BION1 (S4; DI 16.23) and PE 40–60 °C (S7; DI 33.71), respectively. The solvents with the strongest effects largely contain either naphtha [Un-Du, Un-Stick i.e. a liquid mixture of hydrocarbons (mainly aliphatic and aromatic hydrocarbons, sulphur compounds)] or petroleum ether (BION1, PE 40–60 °C i.e. mainly aliphatic hydrocarbons) (Table 1). No direct genotoxic or DNA-damaging effect has been reported for these listed major constituents. Furthermore, these (or at least similar) components are also listed for solvents (S400, “Petrol mixture”, WD-40) where no increased DI was measured. Therefore, we are currently unable to explain the observed differences in solvent effects. However, (unlisted) minor components or impurities could also play a role. In contrast to “dry exposure”, where the overall effects on DNA recovery were milder and did not differ significantly between solvents and the water control, the effects of “wet exposure” were more variable, and for certain solvent samples, losses were much more pronounced in terms of quantity and quality.

Moisture generally affects DNA stability by promoting microbial activity and providing a substrate for hydrolytic reactions [41]. Under ordinary physiological conditions or in vitro in aqueous solution, DNA forms a double helical structure which is relatively stable because of the stacking of the amine bases and of the hydrogen bonding between them [42]. The addition of a suitable organic co-solvent can reduce the thermal stability of base pairing and thereby improve the efficiency of PCR and DNA sequencing [29]. Further increased concentration of the co-solvent may lead to “denaturation” of the double helix and transition to disordered single-stranded coils, which also depends on factors such as temperature, DNA concentration, pH and salt concentration [43]. The single-stranded DNA is more prone to damage than the double-stranded helix, due to greater exposure of chemically reactive moieties in the amine bases [44]. Thus, it was expected that the stability of DNA in solution over time would be less than in the dry state at room temperature.

Notably, there are differences in the water solubility of the adhesive removers studied, with the solvents that appear to have the strongest effects on DNA stability being largely insoluble and non-polar (Table 1). Non-polar organic liquids are poor solvents for DNA. Transfer of DNA from aqueous solution to a non-polar solvent is energetically unfavourable and occurs with the hydrated DNA encapsulated inside micro-droplets of water [45]. Depending on the type (water solubility) and amount of organic solvent, nucleic acid concentration (or availability of suitable carrier), incubation conditions (time and temperature) and particularly sufficient cations to neutralize negative charges on the phosphate backbone, compact DNA precipitates may be formed [43]. However, the experimental conditions for the “wet exposure” were such that efficient DNA precipitation was rather unlikely (e.g. salt concentration too low). It is more likely that multiple factors (e.g. osmotic pressure, dielectric constant, specific interactions with nucleic acid strands and non-canonical DNA structures) have contributed to the observed DNA degrading effects and differences between solvent treatments [43].

Several studies have recommended a direct PCR approach for STR analysis of latent minimal traces (latent “touch” and fingerprints) in order to avoid the loss of initial template during extraction and to reduce the risk of contaminations [46, 47]. However, this method bears an increased risk of PCR inhibition due to impurities in the crude template preparations, and efficient amplification is particularly important in the context of casework-type samples (low quality and quantity of DNA). Even with optimal template input, a relatively small amount of “Turkish solution” was sufficient for locus-specific drop-outs or even complete failure of amplification during STR profiling with the NGM Detect™ Kit (Supplementary Fig. 2c–e), and even more pronounced effects can be expected with compromised template. Therefore, it is important to point out that the IPC *C*_*T*_ values of all test samples of the “wet exposure” (same as for the “dry exposure”) were not elevated, compared to the controls and quantification standards, confirming that silica-based DNA purification can be quite efficient in removing inhibitory components of the adhesive removers (including S400, “Turkish solution” and WD-40).

### Effect assessment of solvent-based adhesive removal on fingerprint development

In order to investigate the relevance of the time interval between the application of a solvent and the physical detachment of an adhesive object from its support, as well as the downstream consequences for the subsequent development of fingerprints, a series of adhesive removal experiments were conducted with prepared “mock evidence” (self-adhesive stamp with a fingerprint on the “sticky side” and affixed to a paper envelope) (Supplementary Fig. 3). To mimic casework, the prepared specimens were first subjected to ninhydrin treatment, which is commonly used to develop fingerprints on porous surfaces. Then a time-series experiment with adhesive removal agents was carried out. Solvents [Un-Du (S1), petroleum ether (PE) boiling point: 60–80 °C, PE 80–100 °C, BION1 (S4), Un-Stick (S3), “Turkish solution” (S6)] were dripped onto the back of the envelope with a pipette and after short time intervals of incubation (5, 10, 15 and 25 s, respectively), the stamps were immediately peeled off. Finally, carbon black powder was applied to visualise latent fingerprints on the adhesive side of each stamp. Fingerprints remained well preserved up to 10 s of solvent treatment before complete detachment, while a treatment of 15 s or longer was apparently destructive to the adhesive layers and the prints present on them. Although some solvents differed considerably in their chemical composition, they caused very similar effects on fingerprint integrity in a time-dependent manner.

Overall, it seems that the solvent treatment alone (in small quantities and under the given experimental conditions) is not sufficient to affect print quality, while direct physical contact with a porous substrate in combination with solvent treatment appears to have a much stronger effect. Detachment of the stamp from the moistened paper led to a rapid termination of the “reaction”, apparently due to the interrupted “suction process”.

### Effect assessment of adhesive removal on DNA recovery

Previous studies have shown that the amount of DNA transferred from its original location to another (transfer between primary and secondary substrate) upon physical contact is largely determined by the surface porosity of both substrates, the condition (dry or moist) of the DNA-containing medium and the intensity of contact [4, 31]. In the following experiments, we investigated the influence of solvent-based adhesive removal on secondary DNA transfer under varying test conditions i.e. different treatment times and locations of initial DNA deposition (a fixed amount of 50 ng DNA applied to (a) the adhesive side of the stamp, (b) the envelope and (c) the non-adhesive side of the stamp, respectively).

**Fig. 3:**
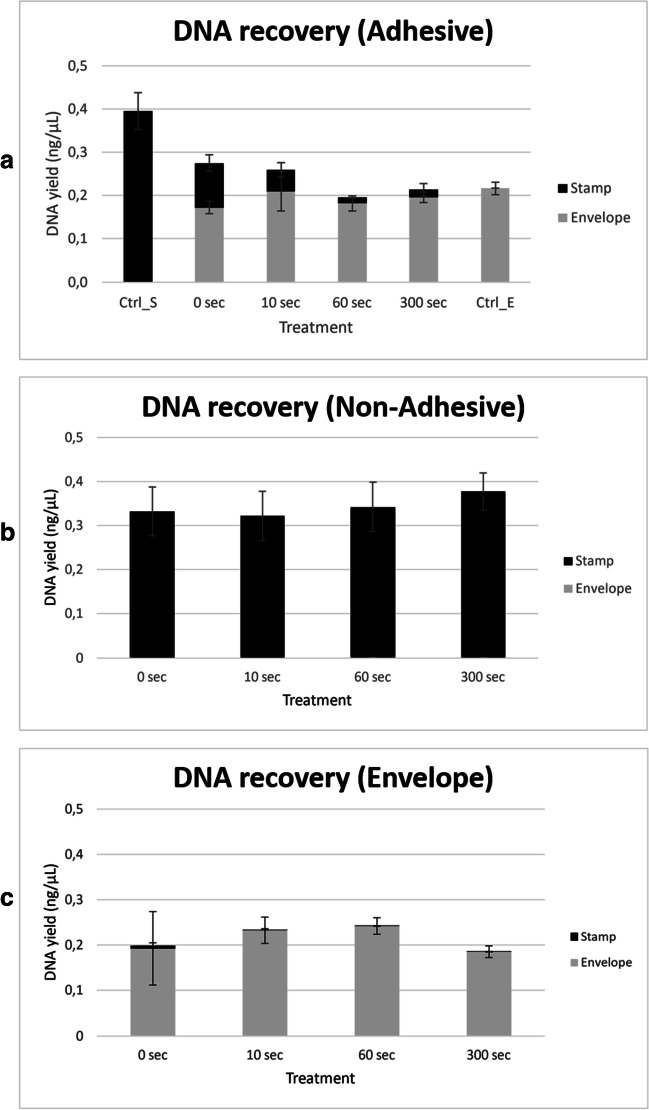
**Solvent treatment via the reverse side removes DNA deposits from the adhesive side of an affixed stamp, but hardly affects DNA deposits on the non-adhesive side or the envelope**. Displayed stacked bar charts were built in MS Excel (2016) with DNA quantitation data from real-time PCR analysis [mean values for the small autosomal target (“DNA yield (ng/µL)”)] in correspondingly treated samples from stamps (black) and envelope (grey) (on the *y*-axis) were plotted against “Treatment” (i.e. time of incubation with “Turkish solution” (S6) prior to physical separation of affixed stamps from envelope plus controls; on the *x*-axis in ordinal scale). Three different types of test specimens were prepared: **a** “Adhesive” (upper panel) i.e. postage stamps with 50 ng DNA applied to the adhesive side and affixed to an envelope; **b** “Non-Adhesive” (middle panel) i.e. postage stamps with 50 ng DNA applied to the non-adhesive side and affixed to an envelope; **c** “Envelope” (lower panel) i.e. envelope treated with 50 ng DNA in marked areas and then sealed with stamps. Prepared specimens were stored overnight (18 h) at room temperature and in the dark. A time-series of solvent-treatment (300 μL of “Turkish solution” applied to the envelope at the stamp’s back-side) prior to the complete physical separation (stamp from envelope paper) was performed and included the following time points: 10, 60 and 300 s and untreated control (0 s). The following controls (without solvent-treatment) were also included: stamps with DNA deposition (Ctrl_S), not affixed to an envelope, and envelope with DNA deposition (Ctrl_E) but no stamp affixed, respectively. Each combination (type of sample/solvent and time point) was tested in duplicate. DNA was isolated from stamps and paper and subjected to quantitation using the Quantifiler HP Kit

For this purpose, adhesive removal was conducted with the aid of “Turkish solution” (S6). Solvent aliquots (300 μL each) were applied by pipetting onto the back of the envelope and incubated for different time intervals (10, 60 and 300 s) until stamps were peeled off. DNA purified from stamps and envelopes was subjected to quantitative real-time PCR analysis (Fig. 3).

To examine the extraction efficiency of our methodology, we prepared substrate controls with DNA deposition i.e. postage stamps (DNA on the adhesive side, but not affixed on an envelope) and pieces of envelope (DNA applied, but no stamp affixed). A one-tailed *t*-test for independent samples on quantitation data of purified DNA samples revealed a significant (*p* ~ 0.001) difference in extraction efficiency between stamps (~40%) and envelopes (~22%) (Fig 3, upper panel and data not shown).

With the adhesive surface of the stamp as the primary substrate, affixing of stamps resulted in a pronounced DNA transfer (~63% of the total DNA recovered) to the envelope as the secondary substrate within 18 h. Recently, Ruprecht et al. [15] reported that a fingerprint is transferred from the adhesive side of a stamp to the envelope within 2 days of deposition in sufficient quality for further analysis for identification purposes. Our observation suggests that DNA behaves similarly, and we hypothesize that the adhesive layer of the stamp serves as a viscous transport medium towards the secondary porous substrate in this process. The total amount of DNA recovered (i.e. both substrates combined) was about 27% of the initial input (Fig. 3, upper panel). Solvent treatment resulted in the rapid loss of DNA from the stamp (presumably related to removal of adhesive layers), while the amount of DNA on the envelope was only slightly reduced. A one-way ANOVA with repeated measures was performed to assess the effects of solvent treatment on DNA yield (ng/μL) from the adhesive side of the stamp and revealed a statistically significant difference (*p* < 0.001; d.f. = 3; *F* = 107.53) between groups (data not shown). Tukey’s HSD test for multiple comparisons found that the mean value of DNA yield was significantly different between untreated (0 s) and each solvent-treated group (10 to 300 s; *p* = <0.001; 95% *CI* = 0.03 and 0.11). There was no statistically significant difference in DNA yield between untreated and solvent-treated envelope (data not shown).

In contrast, using the envelope as primary DNA substrate (Fig. 3 (lower panel)) hardly resulted in DNA transfer (~3% of the total DNA recovered) to the adhesive surface of an affixed clean stamp as secondary substrate. Solvent treatment caused the rapid loss of DNA from the stamp, while the amount of DNA on the envelope was not significantly affected. The average total amount of DNA recovered (i.e. both substrates combined) was about 22% of the initial input. With the non-adhesive side of the stamp as primary DNA substrate and the stamp affixed to the envelope (Fig. 3 (middle panel)), application of solvent to the back of the envelope had no significant impact on DNA recovery. Furthermore, no DNA transfer was observed, indicating that the backing material of the stamp is permeable to the solvent but not to DNA. The average amount of DNA recovered was about 34% of the initial input.

Paper has previously proved to be a considerably challenging porous substrate for recovering DNA [48–50]. Purification of nucleic acids with filter paper is mainly associated with cellulose, which appears to have similar properties to silica-based materials in binding nucleic acid precipitates under chaotropic conditions [51]. Both DNA and cellulose are amphiphilic macromolecules that have significant polar and non-polar parts suggesting a delicate balance between hydrophilic and hydrophobic interactions [52]. There are few reports that investigated the interaction between cellulose and nucleic acids [51, 53], and there is an obvious need for improved protocols for the purification of native DNA from paper.

Overall, our findings are in agreement with the works of van Oorshot et al. [4], demonstrating that the type of substrates/surfaces that come into contact affects how much DNA is transferred. The authors showed that less DNA is transferred from a porous substrate than from a non-porous primary substrate, while a porous secondary substrate facilitates transfer from the primary substrate [31]. With biological material in its liquid form (compared to its dry state) an increased transfer to a porous substrate was reported, but less transfer from a primary porous substrate to a secondary substrate (irrespective of substrate type). In addition, significant differences in DNA recovery from various porous and non-porous materials were observed [31, 54].

### Effect assessment of combined adhesive removal and fingerprint development on subsequent DNA recovery

Many fingerprint development chemicals and procedures have been tested for their effects on subsequent DNA profiling, and depending on the type of initial sample, the substrate of deposition, and the details of sample processing, sometimes significant losses in DNA quantity and quality have been observed [13]. To better assess the impact of adhesive removal relative to conventional dactyloscopy on DNA recovery, mock evidence (stamps with DNA deposition, affixed to an envelope) was prepared and subjected to chemical fingerprint enhancement with and without an additional step of adhesive removal.

**Fig. 4:**
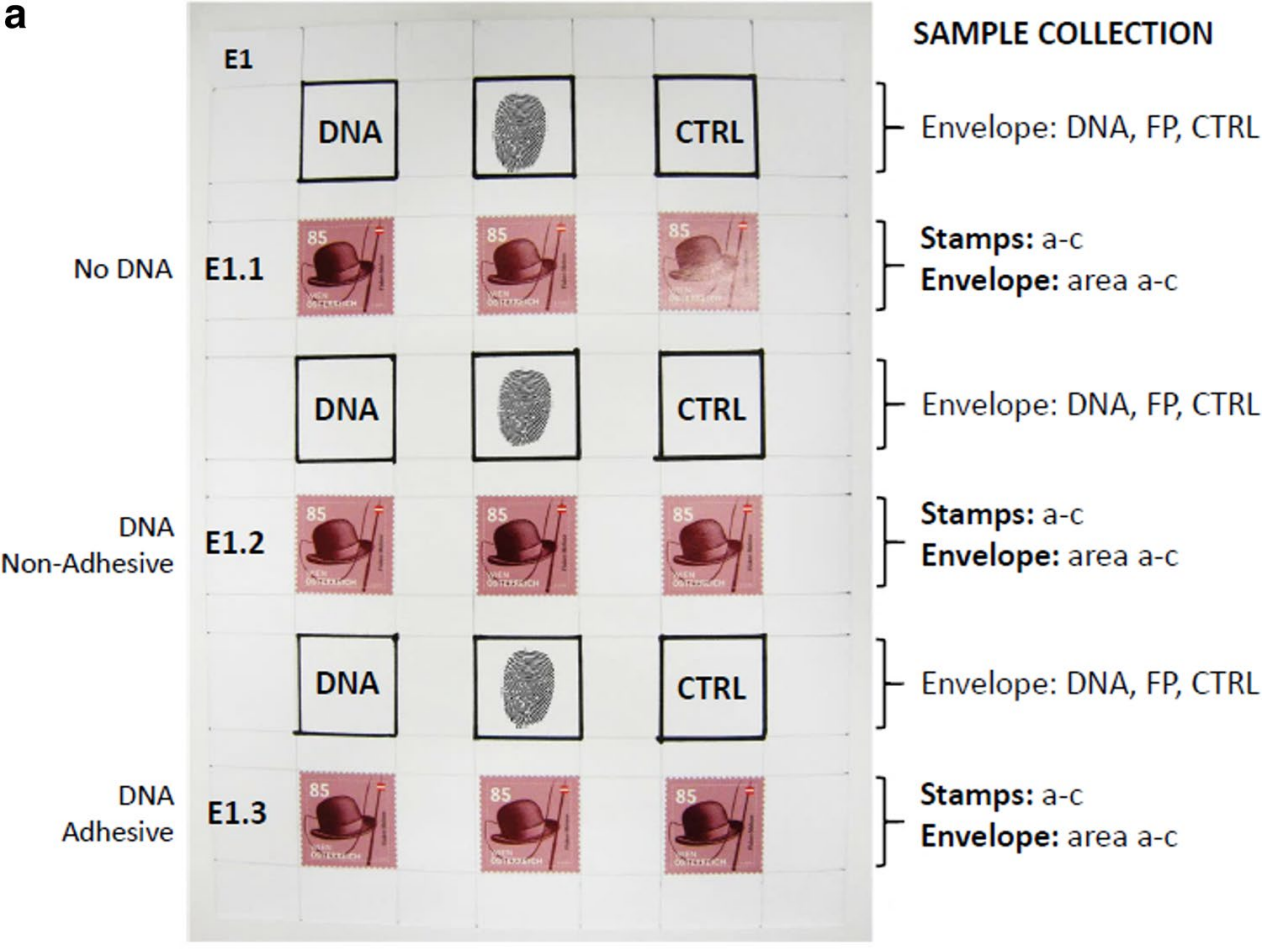

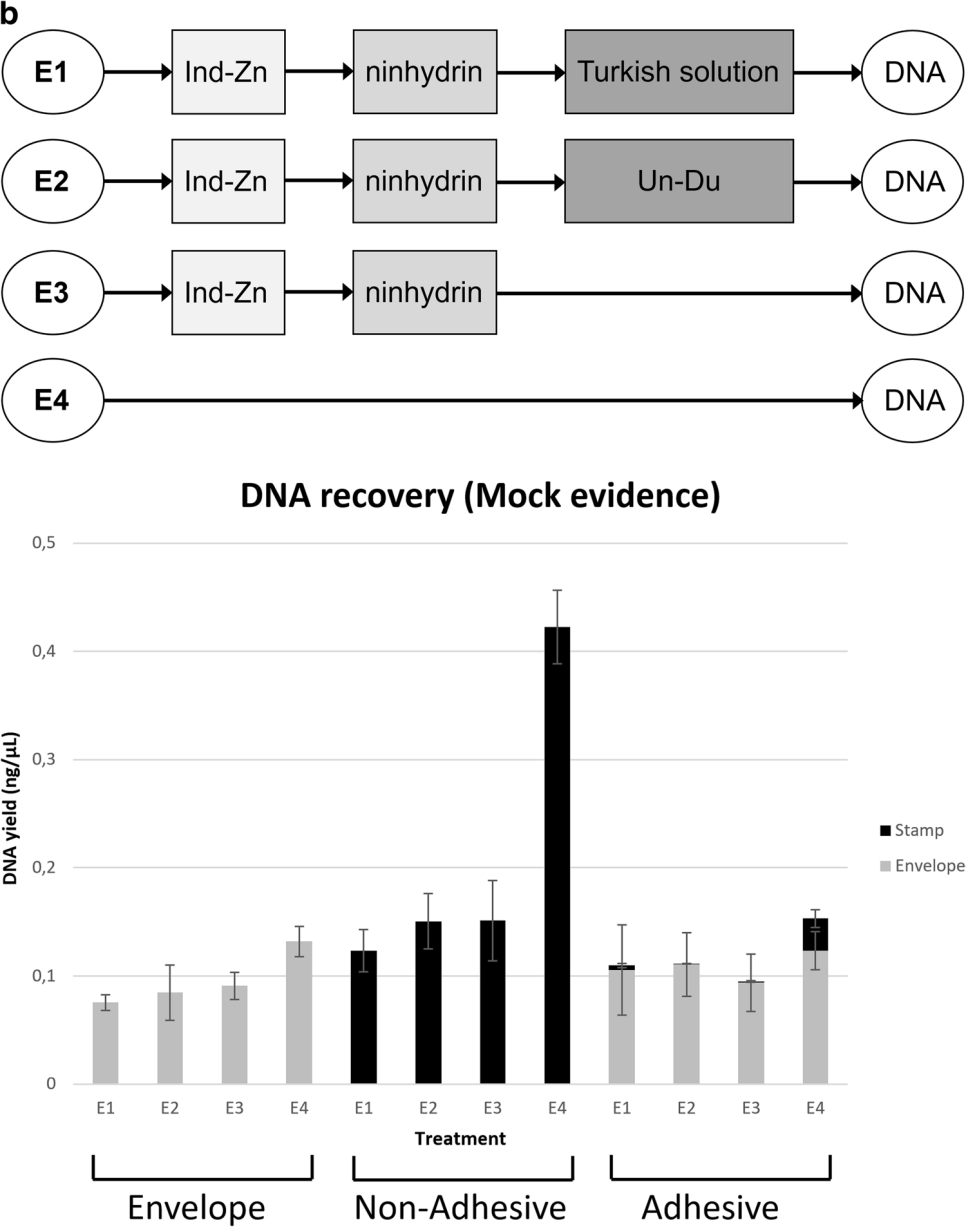
**a** **Preparation and processing of mock evidence for the assessment of combined effects of dactyloscopy and adhesive removal on DNA recovery**. Shown is the basic scheme for the preparation of mock evidence (four envelopes, E1 to E4). Nine square areas (25 × 25 mm^2^) were drawn (in three rows of three squares each) on each envelope and treated (from left to right) with DNA (50 ng each), fingerprint (FP) and untreated (CTRL), respectively. In addition, nine self-adhesive postage stamps were affixed to each envelope in three rows of three. The upper row of stamps served as a control (No DNA). In the second row, DNA was applied to the non-adhesive side of the stamps (DNA Non-Adhesive). In the third row, the stamps were first treated with DNA on the adhesive side and later affixed to the envelope (DNA Adhesive). Prepared envelopes were stored at room temperature in the dark for 1 week. **b** **Flow chart providing an overview on treatments of mock evidence (envelopes E1 to E4)**. Three envelopes (E1 to E3) were processed first using 1,2-indanedione-zinc (Ind-Zn), followed by ninhydrin treatment and incubation in a climate chamber for 48 h. Then the envelopes were cut open laterally. Removal of the stamps was performed either with the aid of adhesive release agents [E1: “Turkish solution“ (S6) and E2: Un-Du (S1)] or purely mechanically using tweezers (E3). Envelope E4 was left untreated (no Ind-Zn/ninhydrin) and stamps were removed mechanically. DNA was isolated from stamps and papers and subjected to quantitation using the Quantifiler HP kit. **c** **Treatment with conventional fingerprint reagents lead to a significant reduction in the amounts of DNA recovered from stamps, while the additional use of adhesive removers did not significantly enhance this effect**. Displayed stacked bar charts were built in MS Excel (2016) with DNA quantitation data from real-time PCR analysis [mean values for the small autosomal target (“DNA yield (ng/µL)”)] in correspondingly treated samples from stamps (black) and envelope (grey) (on the *y*-axis) were plotted against “Treatment” (mock evidence (envelopes E1 to E4) was prepared and processed, as described in Fig. 4a, b). The impact of each treatment was tested in triplicate. Results from testing three different sample types collected from mock evidence are shown here: “Envelope” i.e. excised areas (25 × 25 mm^2^) from envelope with 50 ng DNA deposition. “Non-Adhesive” i.e. postage stamps, each with 50 ng DNA applied to the non-adhesive side and affixed to the envelope; “Adhesive” i.e. stamps treated with 50 ng DNA on the adhesive side and later affixed to the envelope

The mock evidence (i.e. four test envelopes with DNA depositions, E1 to E4) are prepared, as described in “Effect assessment of combined adhesive removal and fingerprint development processes on subsequent DNA recovery from mock evidence (postage stamps attached to envelope” and Fig. 4a. The flow chart in Fig. 4b provides an overview of the different treatments applied. Three envelopes (E1 to E3) were processed first with 1,2-indanedione-zinc (Ind-Zn), followed by ninhydrin treatment. Then the stamps were detached either with the aid of adhesive removers (E1: “Turkish solution” and E2: Un-Du) or purely mechanically using tweezers (E3). Envelope E4 was left untreated (no Ind-Zn/ninhydrin) and stamps were removed mechanically without the application of solvents. DNA recovered from stamps and paper was subjected to quantitative analysis. DNA quantitation data were evaluated using MS Excel (2016) (Fig. 4c).

A significant difference was found between the treatment groups in the amounts of DNA recovered from traces applied to defined areas of the envelope (ANOVA *p* ~ 0.01; *d*.*f*. = 3; *F* = 7.07). Tukey’s HSD test revealed weak or moderately significant differences in the average DNA yield from stamps between the controls (E4) and the samples subjected to fingerprint development (E1 to E3) (*p* ≤ 0.05; 95% *CI* = −0.001 and 0.099).

With the DNA-treated non-adhesive side of stamps as the primary substrate, a significant difference was found between groups in the amounts of DNA recovered from the stamps (ANOVA *p* < 0.001; *d*.*f*. = 3; *F* = 66.87). Tukey’s HSD test gave a statistically significant difference in the average DNA yield from stamps between the controls (E4) and the samples subjected to fingerprint development (E1 to E3) (*p* < 0.001; 95% *CI* = 0.24 and 0.47). The average amount of DNA recovered in the controls was about 52% of the initial input. No human DNA was detected in the envelope within areas of stamp attachment, indicating that no transfer had occurred. Again, adhesive removal had no significant effect on the DNA yield of treated groups.

In untreated controls (E4), affixing of the DNA-treated adhesive surface of a stamp as the primary substrate resulted in substantial DNA transfer (~81 % of the total DNA recovered) to the envelope as the secondary substrate. The total amount of DNA recovered (i.e. both substrates together) in the controls was about 15% of the initial input after 1 week of storage at room temperature. There was no significant difference in total DNA recovery between controls (E4) and treated samples (E1 to E3). However, a significant difference between groups was noted when focusing on the DNA yield obtained solely from the adhesive side of stamps (ANOVA *p* < 0.001; *d*.*f*. = 3; *F* = 32.09). Tukey’s HSD test for multiple comparisons found a statistically significant difference in the average DNA yield between the controls (E4) and the samples subjected to fingerprint development (E1 to E3) (*p* < 0.001; 95% *CI* = 0.01 and 0.04). Importantly, additional adhesive removal (Un-Du/“Turkish solution”) had no significant effect on the DNA yield of the treated groups.

Our investigations with DNA traces on different substrates (adhesive and non-adhesive surface of postage stamp and paper envelope) have repeatedly shown that treatment with fingerprint reagents can lead to significant losses in DNA recovery, while the additional use of adhesive removers (Un-Du, “Turkish solution”) has no pronounced effect. Previous studies have indicated that treatment with ninhydrin may result in quantitative DNA loss, but this often has little or no impact on STR profiling [13, 55]. 1,2-Indanedione/zinc treatment can be very effective in fingermark visualisation with porous surfaces as a single process and even more as a process sequence in combination with ninhydrin [56]. However, deleterious effects of Ind/Zn treatment on DNA have also been reported, in which the substrate type and the time interval between treatment and DNA extraction might play a role [13]. Tsai et al. [57] demonstrated that differences in the Ind-Zn formulation (acidic or neutral i.e. either acetic acid or ethyl acetate–based) can determine whether DNA losses (quantity and quality) occur or not. Notably, the amount of acetic acid in the Ind-Zn working solution used in our present study was very low (see “Effect assessment of combined adhesive removal and fingerprint development processes on subsequent DNA recovery from mock evidence (postage stamps attached to envelope)”), and previous field studies in Germany (Bundeskriminalamt, Wiesbaden) have shown that this formulation does not preclude qualitative DNA analysis [58].

Overall, in our experimental set-up, particularly strong losses in DNA quantity occurred with deposits on the non-adhesive side of a stamp, whereas DNA deposits on the adhesive side or the envelope were much less affected. It can be assumed that the relatively “wet” treatments with ninhydrin or Ind-Zn lead to greater losses simply due to “washing out” processes, especially for deposits from the smooth surface of the non-adhesive side. On the one hand, this may be due to the fact that the working solutions of ninhydrin and Ind-Zn both contain the solvent petroleum ether (PE 40–60 °C) as a major component, and the envelopes were soaked with it during treatment. On the other hand, we have found that the backing material of the stamp may be permeable to the solvent but not to DNA (discussed in “Effect assessment of adhesive removal on DNA recovery”), thus preventing DNA depositions on the non-adhesive side from being absorbed by the envelope paper attached underneath. Accordingly, the additional step of adhesive removal (applying small amounts of solvent to the back of the affixed stamp) is expected to have only a minor effect in comparison.

## Conclusions

The aim of this study was to investigate the impact of solvent-based adhesive removal procedures on some critical steps of forensic DNA analysis, focusing on DNA recovery, transfer and amplification by PCR. We found that some of the solvents tested (“Turkish solution”, S400 and WD-40) interfere with amplification by PCR. Therefore, it seems essential to purify the DNA or at least let the volatile solvents evaporate after use (e.g. before direct PCR). The importance of solvent evaporation was further demonstrated by the observation that hindrance can be detrimental to DNA stability and recovery.

The factors influencing the quality of fingerprint visualisation are multifaceted, and adhesive surfaces are difficult substrates because of their diversity. If the separation cannot be easily performed (e.g. by pulling) due to special material properties, storage conditions and deterioration, one is well advised to carefully select the appropriate procedure. For decision support, the supposed techniques should be initially tested on a small area of the adhesive evidence (if possible) or on reference material of similar quality.

We observed that the time interval between the start of solvent treatment and complete detachment of an affixed postage stamp from paper is essential to the fingerprint quality on the adhesive surface. In our hands, a time interval of 10 s appeared to be optimal. Ideally (if possible), we recommend that two lab workers should collaborate at evidence processing during adhesive removal. Beforehand, the envelope should be cut open so that the back of the paper to which the stamp adheres becomes accessible. First, a corner of the stamp should be selected and lifted using a pair of tweezers. A small amount of the removal solution should be applied directly to the paper on the reverse side to the affixed stamp. While the solution is being absorbed by the substrate, the stamp is pulled with a pair of tweezers starting from one corner (after about 5 s of solvent treatment) until it gets fully detached (ideally about 10 s after the onset of solvent treatment).

Solvent treatment quickly dissolves adhesive layers and mobilizes the contained DNA, resulting in the rapid transfer from a non-porous (stamp) to a porous substrate (envelope). For optimum yield, it would still be important to sample both substrates, although the greater proportion of DNA would be expected with the porous substrate. For interpretation of the evidence regarding the site of primary deposition, solvent-induced transfer always has to be considered.

In summary, we conclude that the use of organic solvents as adhesive removers should rather be avoided in forensic examinations, especially if this treatment is not required and later analyses of fingerprints and/or DNA are planned. However, if separation by pulling proves difficult, adhesive removers are a very effective way of detaching with ease. Finally, if recommendations are being followed (see above), the risk of solvent-based adhesive removal for subsequent fingerprint visualisation can be significantly reduced and appears relatively low for DNA analysis compared to the potential (quantitative) impact of some conventional fingerprint reagents.

## Supplementary information

ESM 1 (4.26 MB)

## Data Availability

The datasets generated during and/or analysed during the current study are available from the corresponding author on reasonable request.
