# Supplementary material for: Effects of solvent-based adhesive removal on the subsequent dual analysis of fingerprint and DNA
Source: Int J Legal Med. 2023 Jul 4;137(5):1373–94. doi: 10.1007/s00414-023-03042-w (PMC10421768; doi:10.1007/s00414-023-03042-w)
Supplement: Supplementary file 1 — ESM 1 (4.26 MB) [file 414_2023_3042_MOESM1_ESM.docx]

**
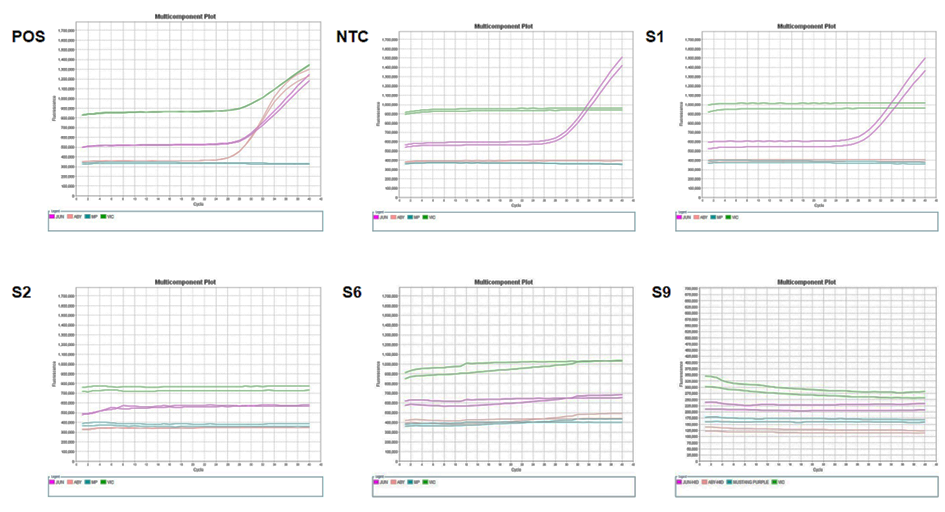
**

**Supplementary Fig. 1. Inhibitor testing using the Quantifiler HP (Human Plus) DNA Quantification Kit.**

Shown are multicomponent plots obtained from real-time PCR analyses using the Quantifiler HP Kit, the 7500 Real-Time PCR System and the HID Real-Time PCR Analysis Software according to the manufacturer's specifications. Fluorescence data (normalised scale, 0 to 1700K) for each target [VIC-labelled human small autosomal (green), ABY-labelled human large autosomal (orange), JUN-labelled **internal PCR control** (**IPC,** **magenta**) and Mustang Purple (MP)-labelled passive reference (blue)] were plotted against cycle number (0 to 40). Displayed are data from duplicate analyses of the following samples (2 µL each): 0.5 ng control DNA (**POS**), no template control (**NTC**), Un-Du (**S1**), S400 (**S2**), “Turkish solution” (**S6**), WD-40 (**S9**).


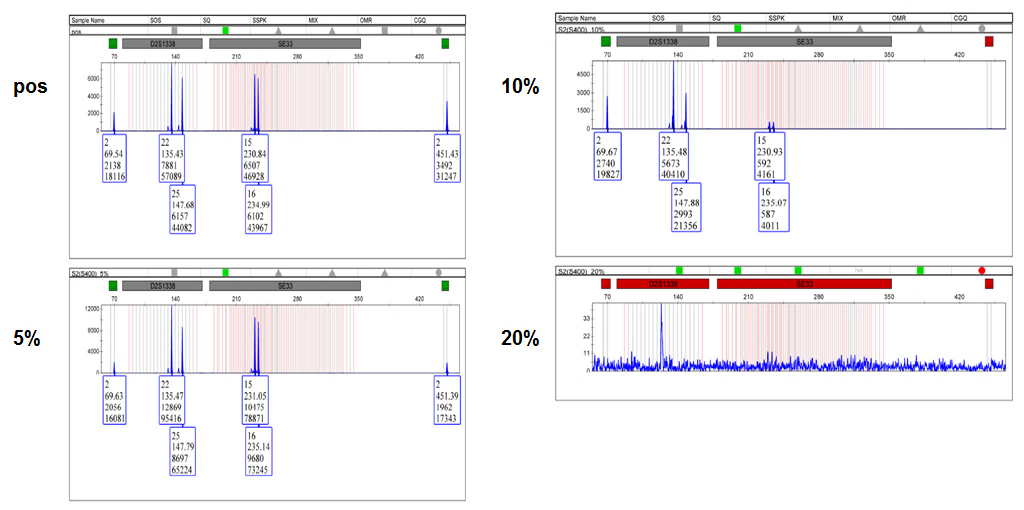


**Supplementary Fig. 2a. Assesment of PCR inhibition by S400 (solvent S2) with the NGM Detect™ kit.**

Partial electropherograms (blue dye channel) of PCR products [6-FAM™-labelled amplicons of the internal quality control markers (**IQCS** (far left) and **IQCL** (far right)) plus two autosomal STR loci (D2S1338, SE33)] obtained with 0.5 ng DNA (2800M Control DNA, Promega), the NGM Detect Kit and in the presence of different amounts [zero (**pos**) to 20% (v/v); in a total reaction volume of 25 µL) of adhesive removal agent S400 (solvent S2) are displayed. PCR products were analysed on the 3500 Genetic Analyzer using GeneMapper ID-X software.


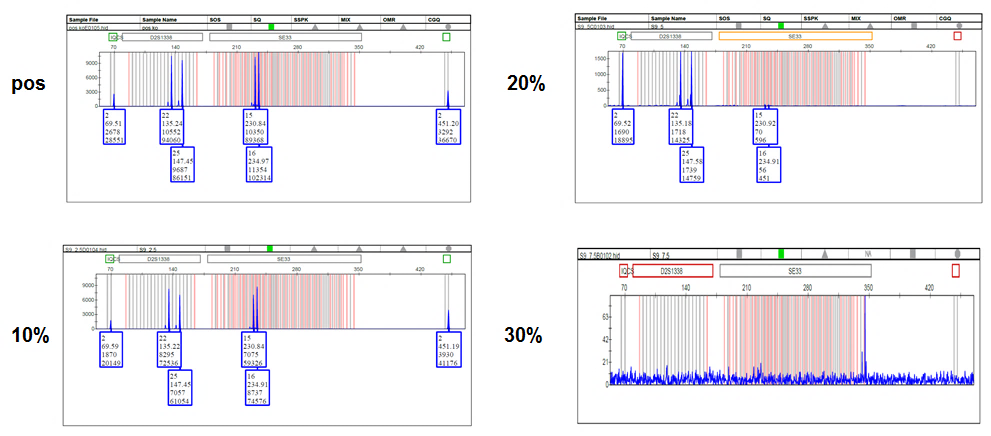


**Supplementary Fig. 2b. Assesment of PCR inhibition by WD-40 (solvent S9) with the NGM Detect™ kit.**

Partial electropherograms (blue dye channel) of PCR products [6-FAM™-labelled amplicons of the internal quality control markers (**IQCS** (far left) and **IQCL** (far right)) plus two autosomal STR loci (D2S1338, SE33)] obtained with 0.5 ng DNA (2800M Control DNA), the NGM Detect Kit and in the presence of different amounts [zero (**pos**) to 30% (v/v); in a total reaction volume of 25 µL) of adhesive removal agent WD-40 (solvent S9) are displayed. PCR products were analysed on the 3500 Genetic Analyzer using GeneMapper ID-X software.


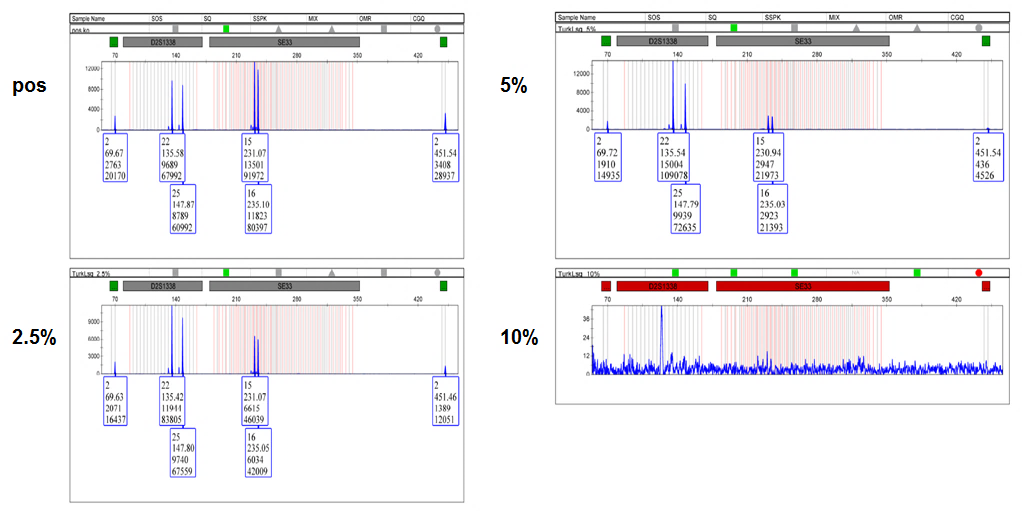


**Supplementary Fig. 2c. Assesment of PCR inhibition by“Turkish solution“ (solvent S6) with the NGM Detect™ kit.**

Partial electropherograms (**blue** dye channel) of PCR products [**6-FAM**™-labelled amplicons of the internal quality control markers (**IQCS** (far left) and **IQCL** (far right)) plus two autosomal STR loci (D2S1338, SE33)] obtained with 0.5 ng DNA (2800M Control DNA), the NGM Detect Kit and in the presence of different amounts [zero (**pos**) to 10% (v/v); in a total reaction volume of 25 µL) of adhesive removal agent“Turkish solution“ (solvent S6) are displayed. PCR products were analysed on the 3500 Genetic Analyzer using GeneMapper ID-X software.


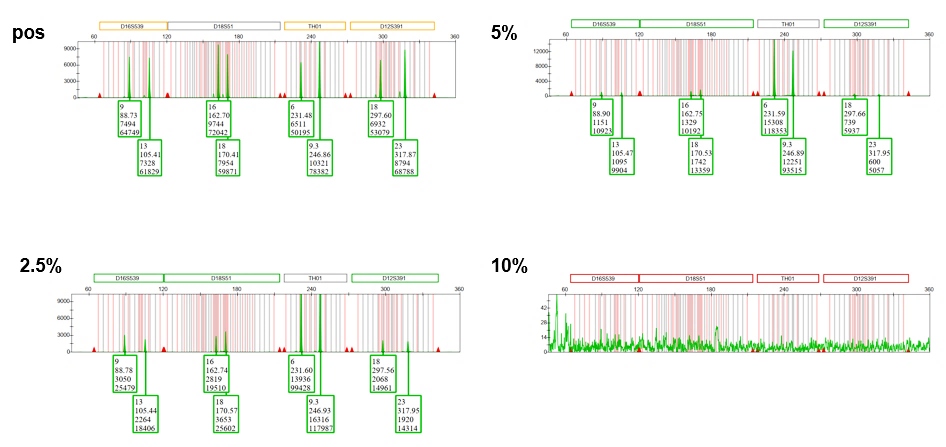


**Supplementary Fig. 2d. Assesment of PCR inhibition by“Turkish solution“ (solvent S6) with the NGM Detect™ kit.**

Partial electropherograms (**green** dye channel) of PCR products [**VIC**™-labelled amplicons of four autosomal STR loci (D16S539, D18S51, TH01 and D12S391)] obtained with 0.5 ng DNA (2800M Control DNA, Promega), the NGM Detect Kit and in the presence of different amounts [zero (**pos**) to 10% (v/v); in a total reaction volume of 25 µL) of adhesive removal agent“Turkish solution“ (solvent S6) are displayed. PCR products were analysed on the 3500 Genetic Analyzer using GeneMapper ID-X software.


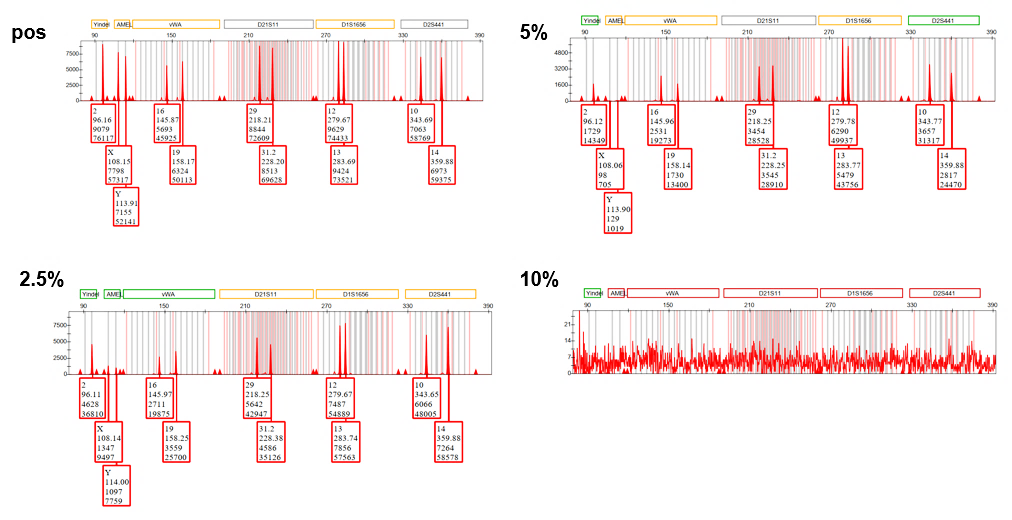


**Supplementary Fig. 2e. Assesment of PCR inhibition by“Turkish solution“ (solvent S6) with the NGM Detect™ kit.**

Partial electropherograms (**red** dye channel) of PCR products [**TAZ**™-labelled amplicons of six genetic marker loci (Y indel, Amelogenin, vWA, D21S11, D1S1656 and D2S441)] obtained with 0.5 ng DNA (2800M Control DNA), the NGM Detect Kit and in the presence of different amounts [zero (**pos**) to 10% (v/v); in a total reaction volume of 25 µL) of adhesive removal agent “Turkish solution” (solvent S6) are displayed. PCR products were analysed on the 3500 Genetic Analyzer using GeneMapper ID-X software.


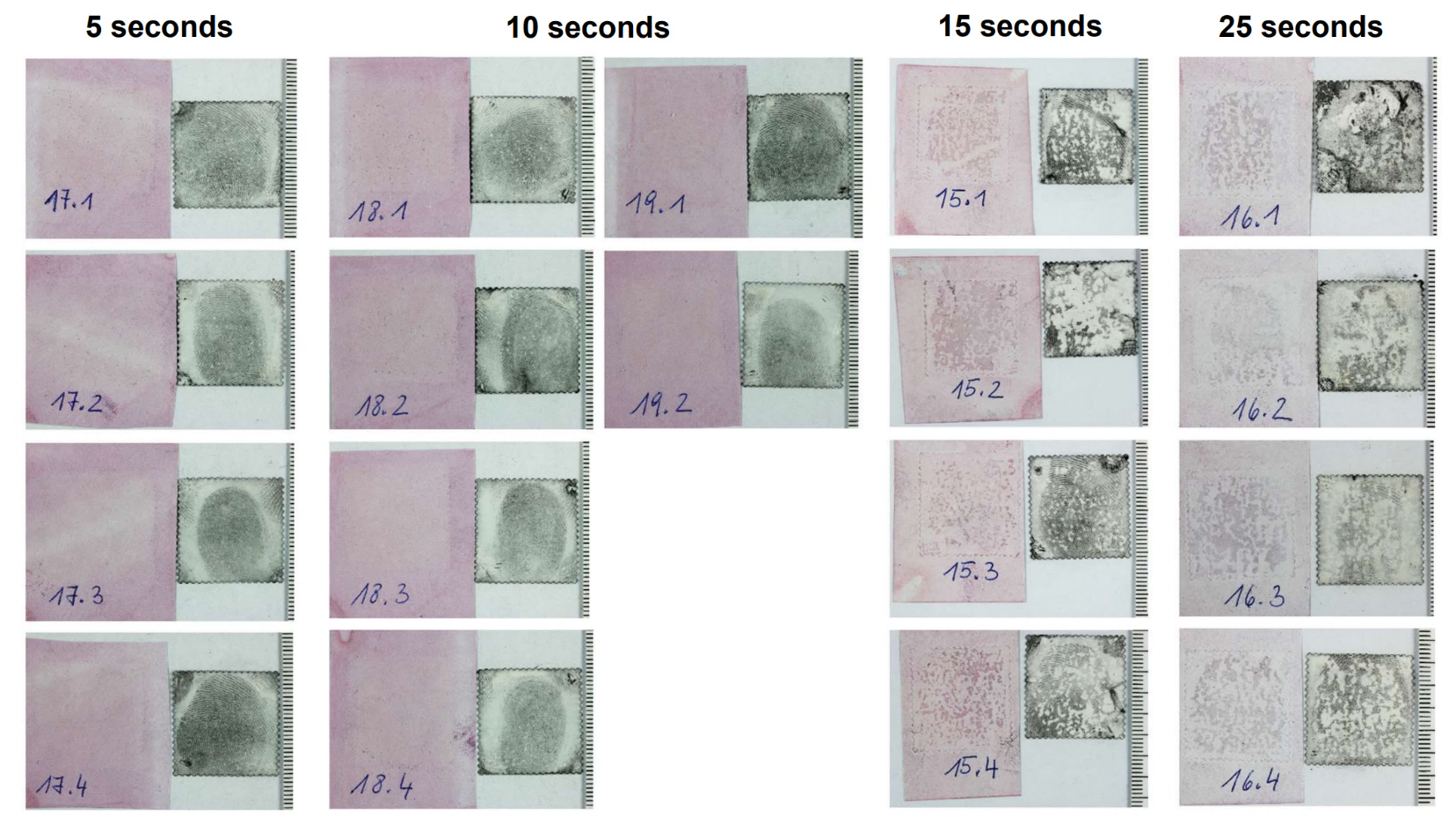


**Supplementary Fig. 3. Effects assesment of adhesive removal processes on the subsequent visualisation of latent fingerprints applied to the adhesive side of postage stamps.**

Prepared specimens (postage stamps labelled with a fingermark on the adhesive side and affixed to an envelope; details provided in 2.4.1.) were first subjected to ninhydrin treatment. Then a time-series experiment with organic solvents was carried out [**five** (samples 17.1 to 17.4), **ten** (samples 18.1 to 19.2)**, 15** (samples 15.1 to 15.4) **and 25 seconds** (samples 16.1 to 16.4)**,** respectively] prior to the physical separation of affixed stamps from the envelope. Finally, carbon black powder was applied to visualise latent fingerprints on the adhesive side of each stamp. The following organic solvents were used as release agents: **Un-Du (S1)** [used with samples **17.1, 18.1, 15.1, 16.1**], **petroleum ether (PE) boiling point: 60-80°C** [samples **17.2, 18.2, 15.2, 16.2**], **PE 80-100°C** [samples **17.3, 18.3, 15.3, 16.3**], **BION1 (S4)** [samples **17.4, 18.4, 15.4, 16.4**], **Un-Stick (S3)** [sample **19.1**]and “**Turkish solution” (S6)** [sample **19.2**].

**Supplementary Table 1a Quantative real-time PCR analysis of DNA recovered from “dry exposure” to organic solvents (S1 to S9).**

MS Excel (2016) was used to calculate mean values based on DNA quantitation data [(small autosomal antigen (**SA**), degradation index (**DI**) and the internal PCR control (**IPC**)] from real-time PCR. Mixtures of human nuclear DNA (control DNA from the Quantifiler HP Kit) and different solvents (organic solvents **S1 to S9** and amplification grade water (Promega) as control (**CTRL**), respectively) were prepared. Aliquots of about 50 µL (each containing 50 ng DNA) were applied to sterile cotton swabs allowing the solvent-DNA mixture to evaporate and dry freely. Time series experiments were performed with the following incubation times: five minutes (5 min), one hour (1 h), 18 hours (overnight, ON) and one week (1 W), respectively. Combinations (type of sample/solvent, time point) were tested in duplicate. Subsequently DNA was purified from swabs and subjected to quantitation using the Quantifiler HP kit.

| **Sample Type** |  | **5 min** | **1 hour** | **ON (18 hours)** | **1 week** |
| --- | --- | --- | --- | --- | --- |
| Solvent (S1-9) | **DNA (ng/µL) ^a^** | 0,39 ± 0,04 | 0,37 ± 0,06 | 0,20 ± 0,03 | 0,19 ± 0,03 |
| CTRL |  | 0,36 ± 0,06 | 0,36 ± 0,06 | 0,27 ± 0,10 | 0,14 ± 0,04 |
|  | | | | | |
| Solvent (S1-9) | **Norm. ^b^** | 1,00 ± 0,11 | 0,94 ± 0,16 | 0,51 ± 0,09 | 0,47 ± 0,08 |
| CTRL |  | 1,00 ± 0,17 | 1,01 ± 0,16 | 0,74 ± 0,26 | 0,38 ± 0,12 |

^a^ Calculated mean values for the small autosomal target (SA) ± standard deviation.

^b^ Normalized quantitation data (i.e SA values devided by the respective SA values at 5 min).

|  |
| --- |

| **Sample Type** | **Degradation Index (DI)** | | | **Internal PCR Control (IPC)** | | |
| --- | --- | --- | --- | --- | --- | --- |
|  | Mean (± STD) | Min | Max | Mean (± STD) | Min | Max |
| Solvents (S1-9) | 0,87 ± 0,11 | 0,62 | 1,08 | 27,77 ± 0,23 | 26,97 | 28,51 |
| CTRL | 0,94 ± 0,18 | 0,80 | 1,19 | 27,85 ± 0,28 | 27,44 | 28,04 |

Descriptive statistical parameters (mean ± standard deviation, minimum, maximum) of values for the degradation index (DI) and the internal PCR control (IPC).

**Supplementary Table 1b Quantative real-time PCR analysis of DNA recovered from “wet exposure” to organic solvents (S1 to S9).**

MS Excel (2016) was used to calculate mean values based on DNA quantitation data [(small autosomal antigen (**SA**), degradation index (**DI**) and the internal PCR control (**IPC**)] from real-time PCR.

Mixtures of human nuclear DNA (control DNA from the Quantifiler HP Kit) and different solvents (organic **solvents S1 to S9** and amplification grade water (Promega) as control (**CTRL**), respectively) were prepared. Aliquots of about 100 µL (each containing 50 ng of DNA) were incubated in closed screw-capped sample tubes (Qiagen), in order to reduce the evaporation of volatile organic compounds. A time series experiment was conducted including the following time points of incubation: five minutes (5 min), one hour (1 h), 18 hours (overnight, ON) and one week (1 W), respectively. Each combination (type of sample/solvent, time point) was tested in duplicate. Subsequently DNA was purified from swabs and subjected to quantitation using the Quantifiler HP kit.

| **Sample Type** |  | **5 min** | **1 hour** | **ON (18 hours)** | **1 week** |
| --- | --- | --- | --- | --- | --- |
| Solvent (S1-9) | **DNA (ng/µL)^a^** | 0,48 ± 0,04 | 0,30 ± 0,11 | 0,19 ± 0,09 | 0,13 ± 0,09 |
| CTRL |  | 0,49 ± 0,12 | 0,46 ± 0,03 | 0,36 ± 0,03 | 0,31 ± 0,07 |
|  | | | | | |
| Solvent (S1-9) | **Norm.^b^** | 1,00 ± 0,08 | 0,63 ± 0,22 | 0,39 ± 0,18 | 0,27 ± 0,19 |
| CTRL |  | 1,00 ± 0,24 | 0,95 ± 0,07 | 0,74 ± 0,06 | 0,64 ± 0,15 |

^a^ Calculated mean values for the small autosomal target (SA) ± standard deviation.

^b^ Normalized quantitation data (i.e SA values devided by the respective SA values at 5 min).

|  |
| --- |

| **Sample Type** | **Degradation Index (DI)** | | | **Internal PCR Control (IPC)** | | |
| --- | --- | --- | --- | --- | --- | --- |
|  | Mean (± STD) | Min | Max | Mean (± STD) | Min | Max |
| Solvents (S1-9) | **3,07 ± 6,86** | 0,62 | **33,71** | 27,85 ± 0,13 | 27,54 | 28,12 |
| CTRL | 0,81 ± 0,06 | 0,74 | 0,87 | 27,98 ± 0,06 | 27,91 | 28,03 |

Descriptive statistical parameters (mean ± standard deviation, minimum, maximum) of values for the degradation index (DI) and the internal PCR control (IPC).
